# Supplementary material for: Cocoa Shell Extract Reduces Blood Pressure in Aged Hypertensive Rats via the Cardiovascular Upregulation of Endothelial Nitric Oxide Synthase and Nuclear Factor (Erythroid-Derived 2)-like 2 Protein Expression
Source: Antioxidants (Basel). 2023 Aug 31;12(9):1698. doi: 10.3390/antiox12091698 (PMC10525428; doi:10.3390/antiox12091698)
Supplement: Supplementary file 1 [file antioxidants-12-01698-s001.zip › antioxidants-2552315-supplementary.pdf]

Supplementary inmunoblot data

Figure S1: Protein expression gels using Western blotting experiments

Heart. Nrf2 total

Gel 1

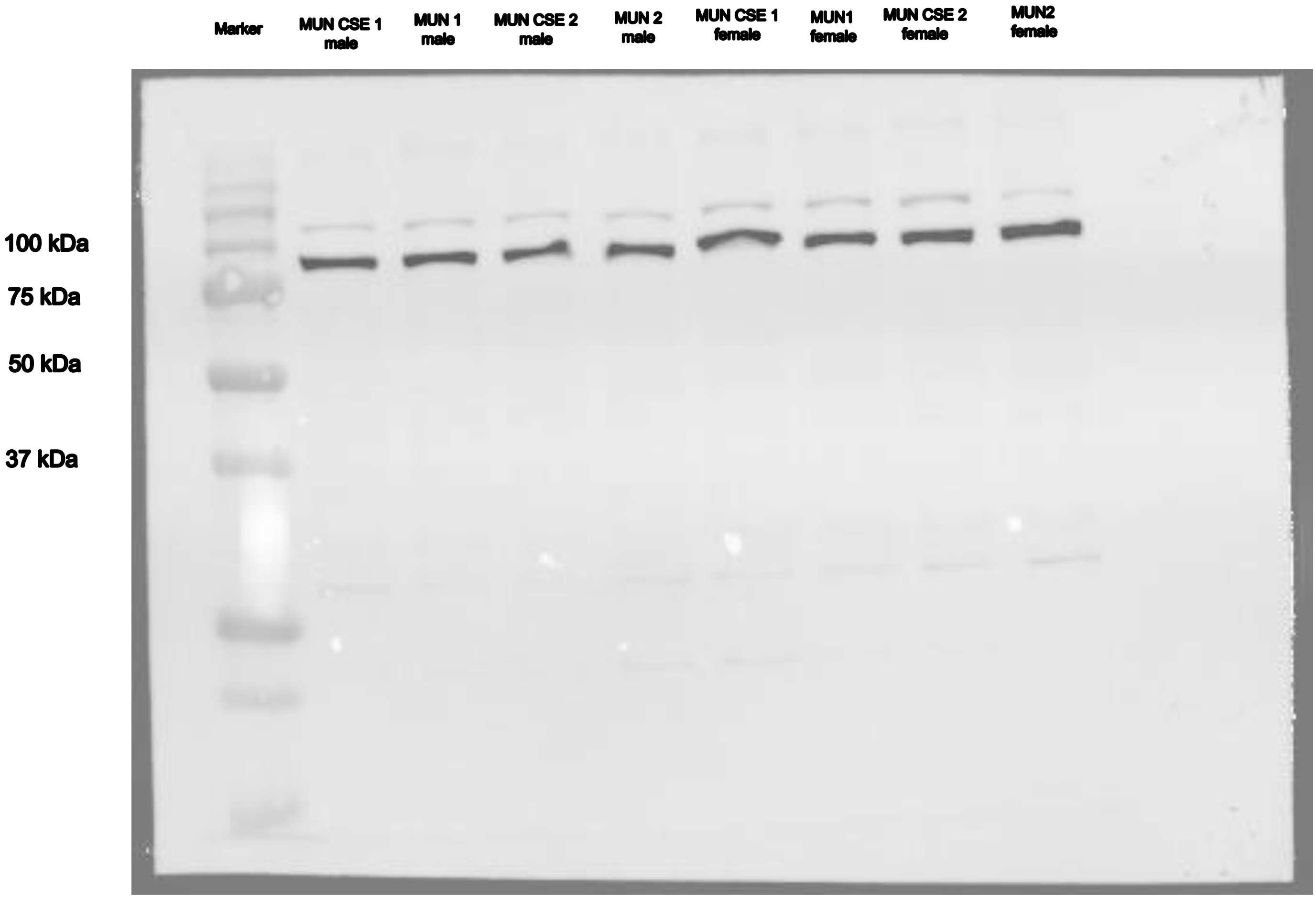

Gel 2

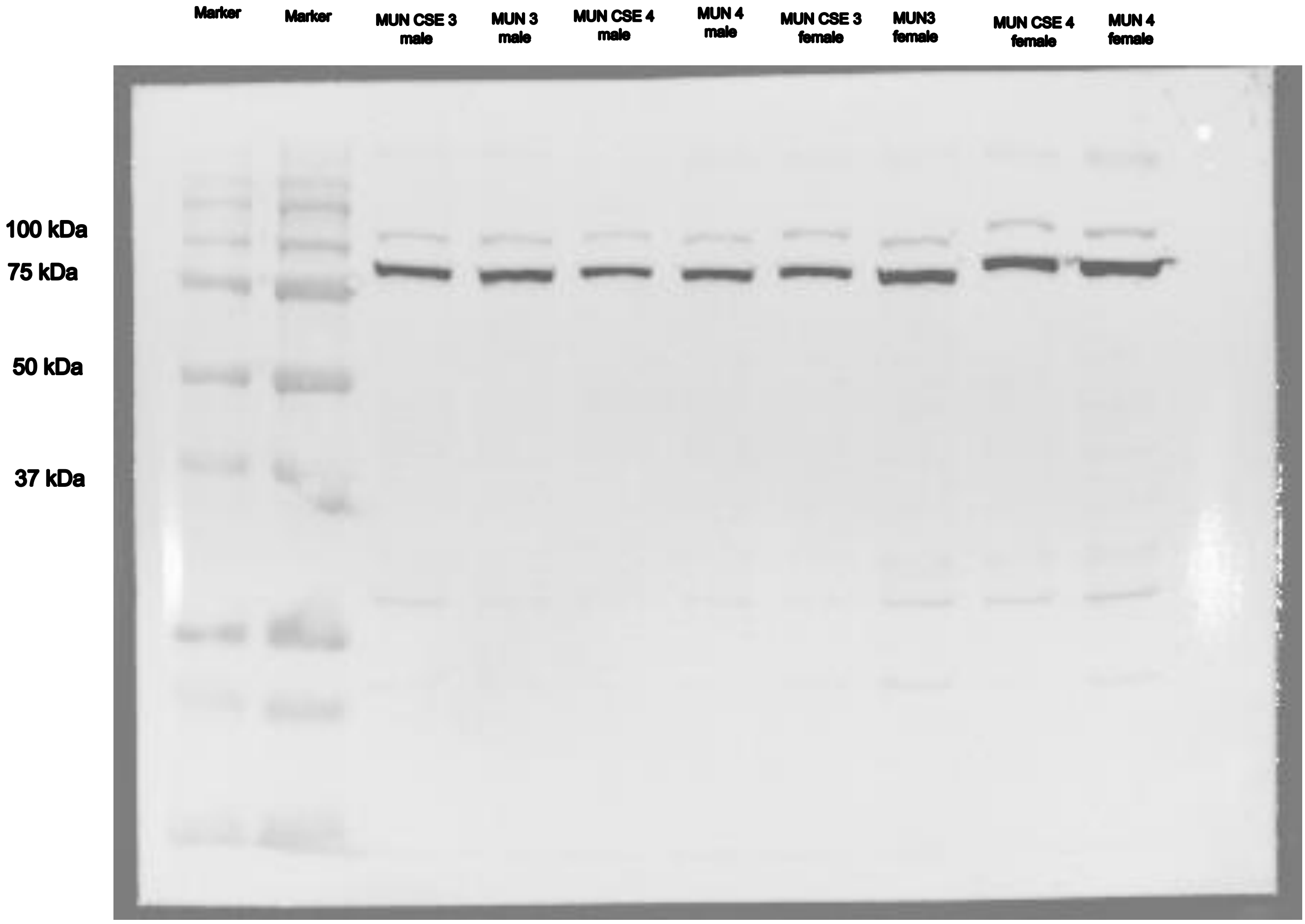
Marker
Marker
MUN CSE 3 male
MUN 3 male
MUN CSE 4 male
MUN 4 male
MUN CSE 3 female
MUN3 female
MUN CSE 4 female
MUN 4 female
100 kDa
75 kDa
50 kDa
37 kDa

Heart. p-Nrf2

Gel 1

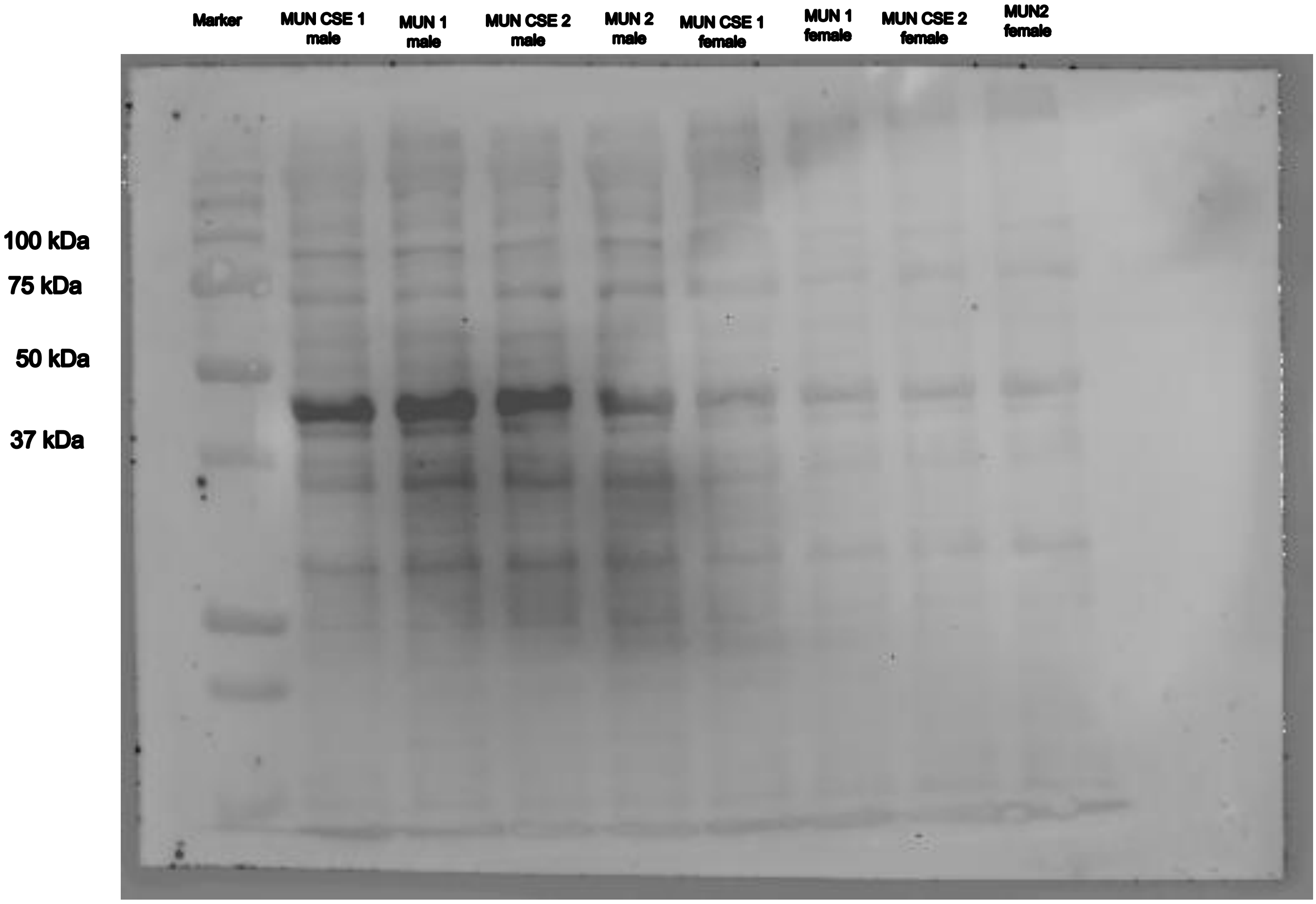

Gel 2

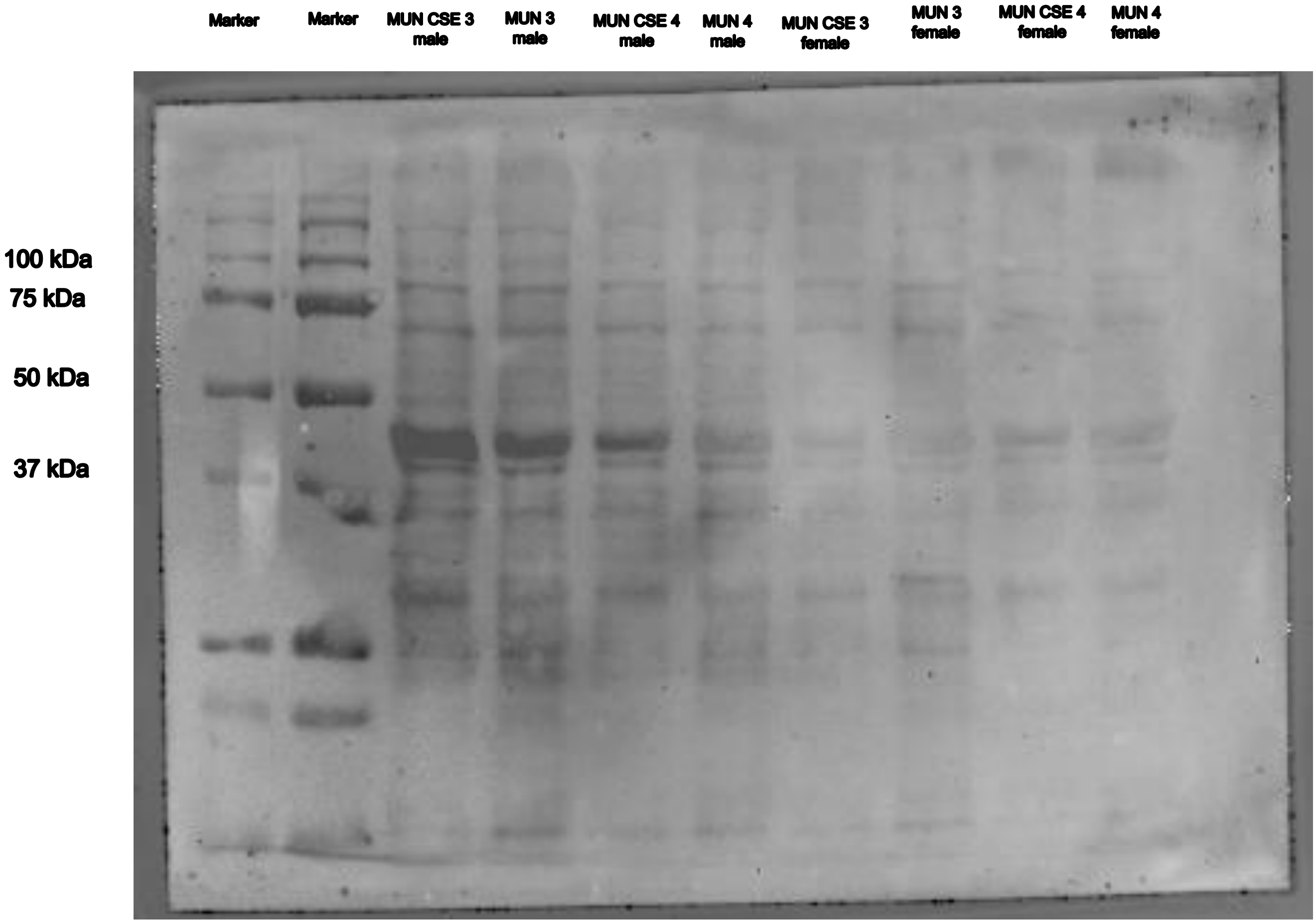
Marker
Marker
MUN CSE 3 male
MUN 3 male
MUN CSE 4 male
MUN 4 male
MUN CSE 3 female
MUN 3 female
MUN CSE 4 female
MUN 4 female
100 kDa
75 kDa
50 kDa
37 kDa

Heart. GADPH

Gel 1

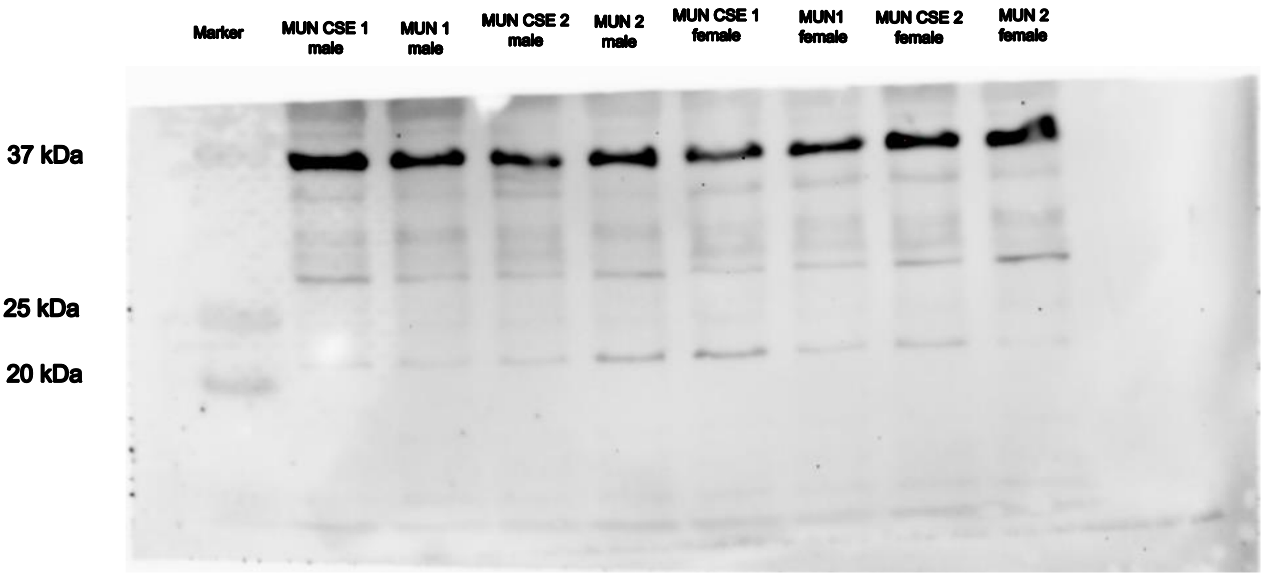

Gel 2

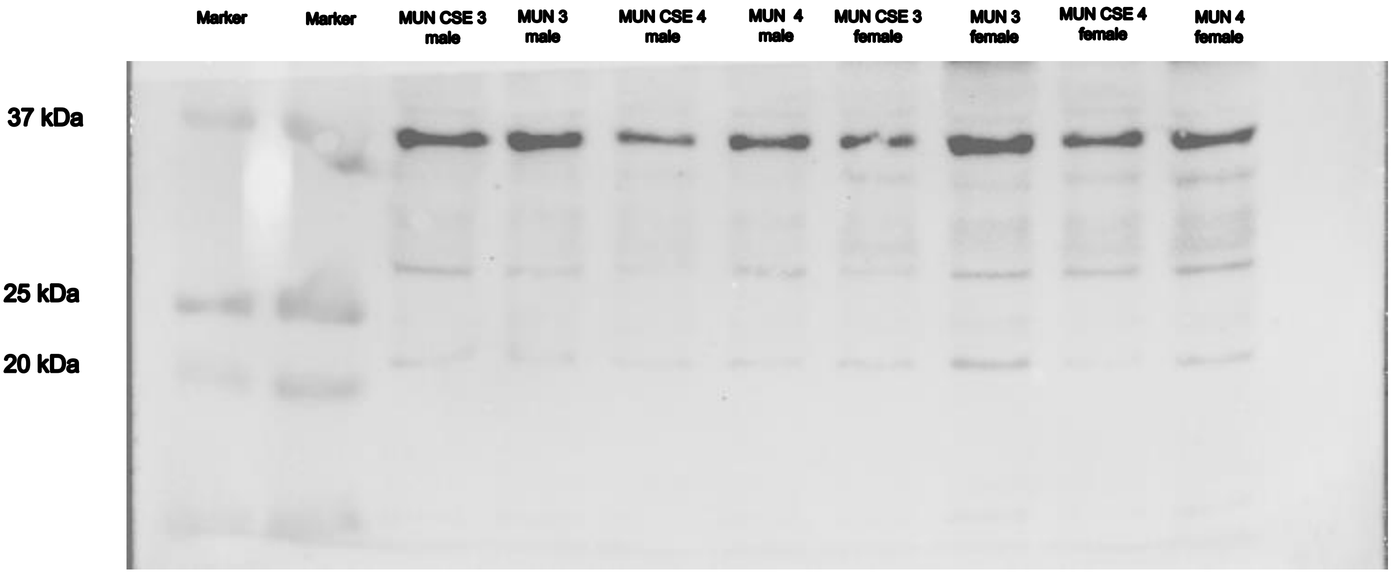

Aorta. Nrf2 total

Gel 3

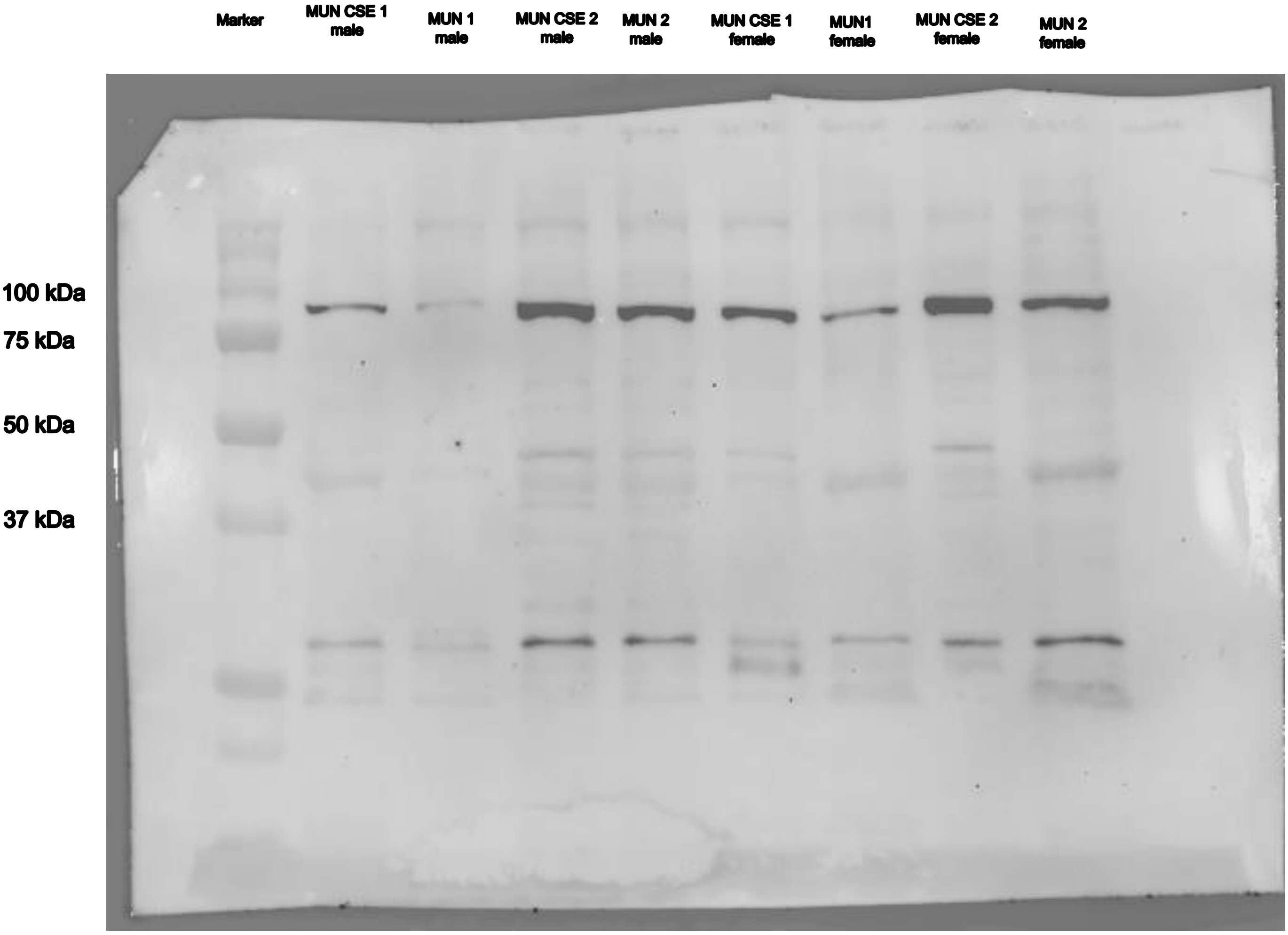

Gel 4

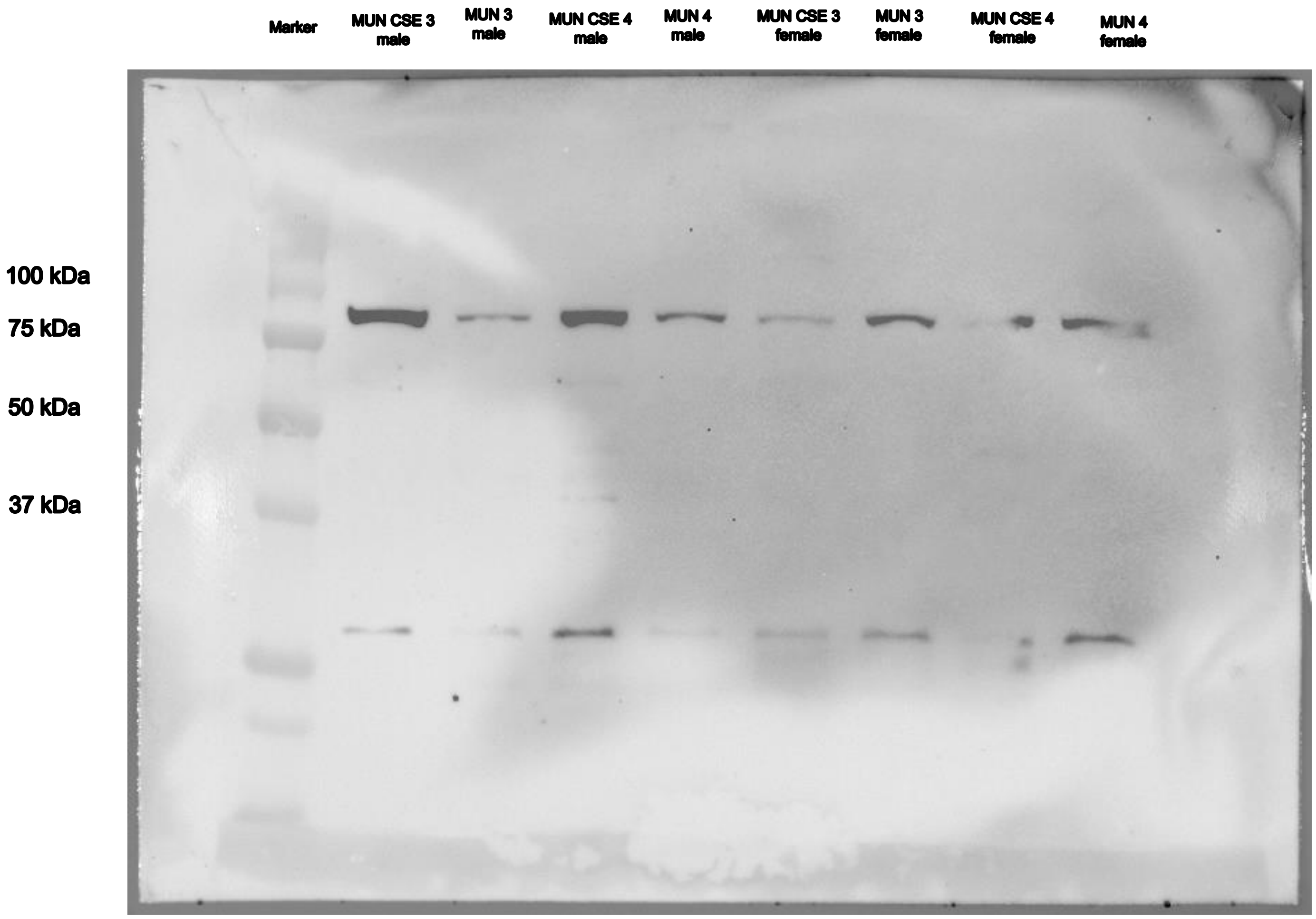
Marker
MUN CSE 3 male
MUN 3 male
MUN CSE 4 male
MUN 4 male
MUN CSE 3 female
MUN 3 female
MUN CSE 4 female
MUN 4 female
100 kDa
75 kDa
50 kDa
37 kDa

Aorta. p-Nrf2

Gel 3

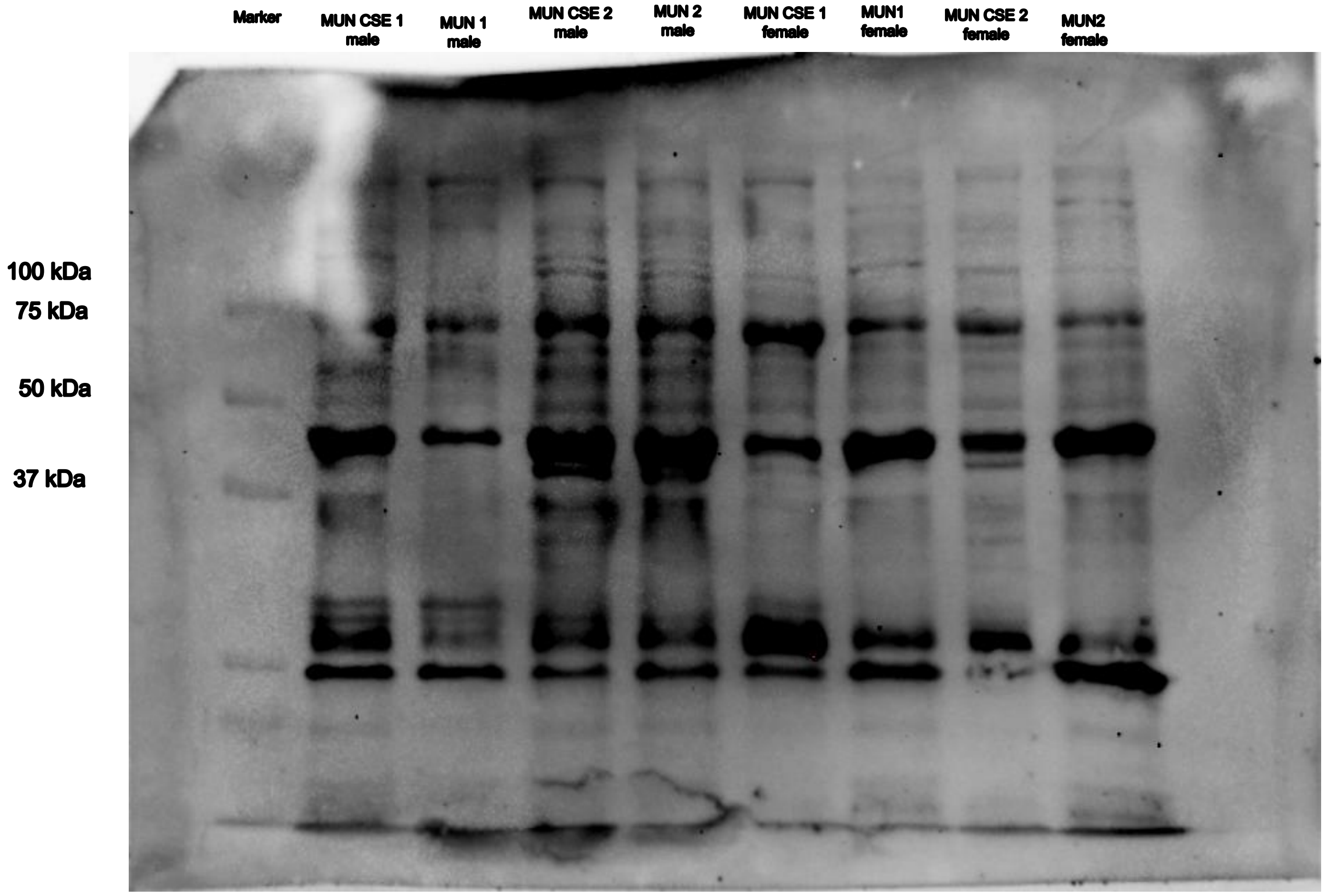

Gel 4

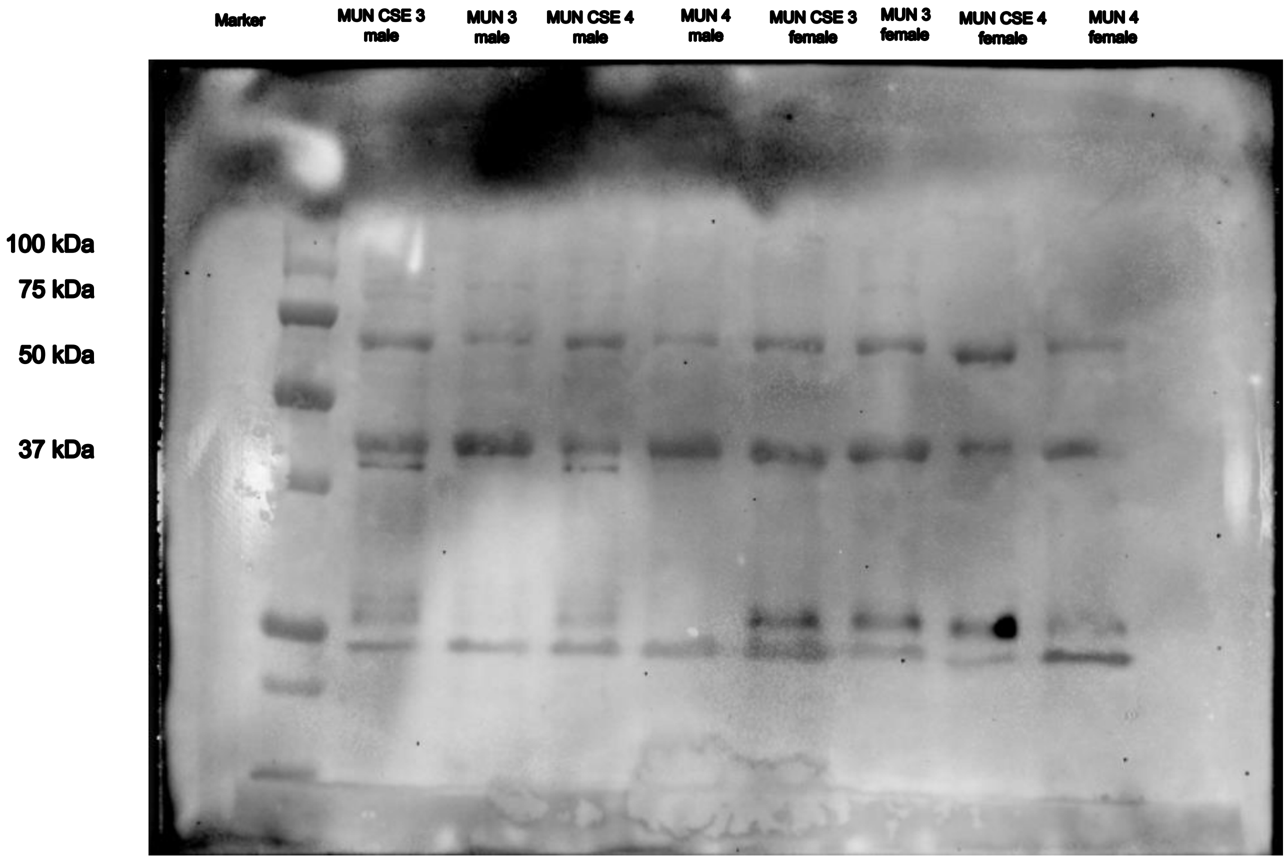
Marker
MUN CSE 3 male
MUN 3 male
MUN CSE 4 male
MUN 4 male
MUN CSE 3 female
MUN 3 female
MUN CSE 4 female
MUN 4 female
100 kDa
75 kDa
50 kDa
37 kDa

Aorta. GADPH

Gel 3

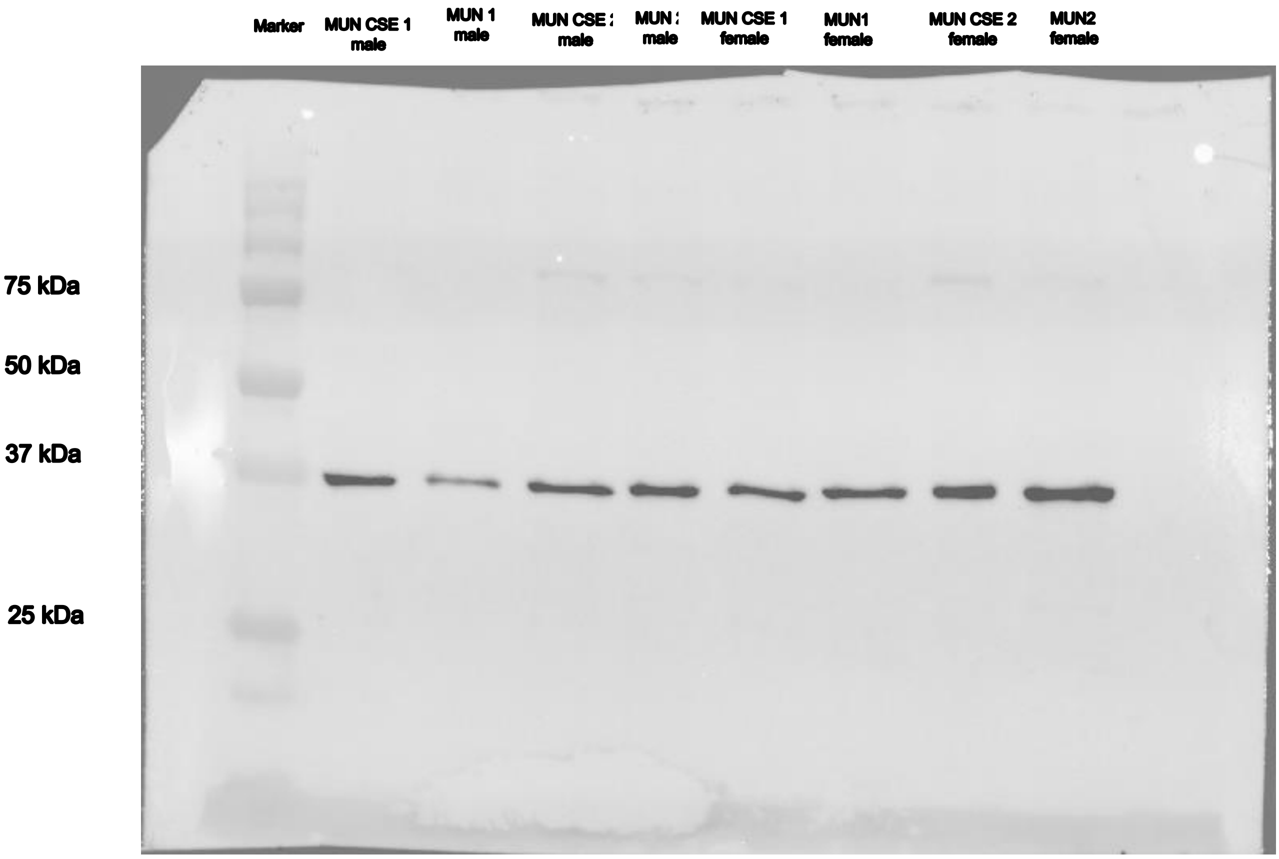

Gel 4

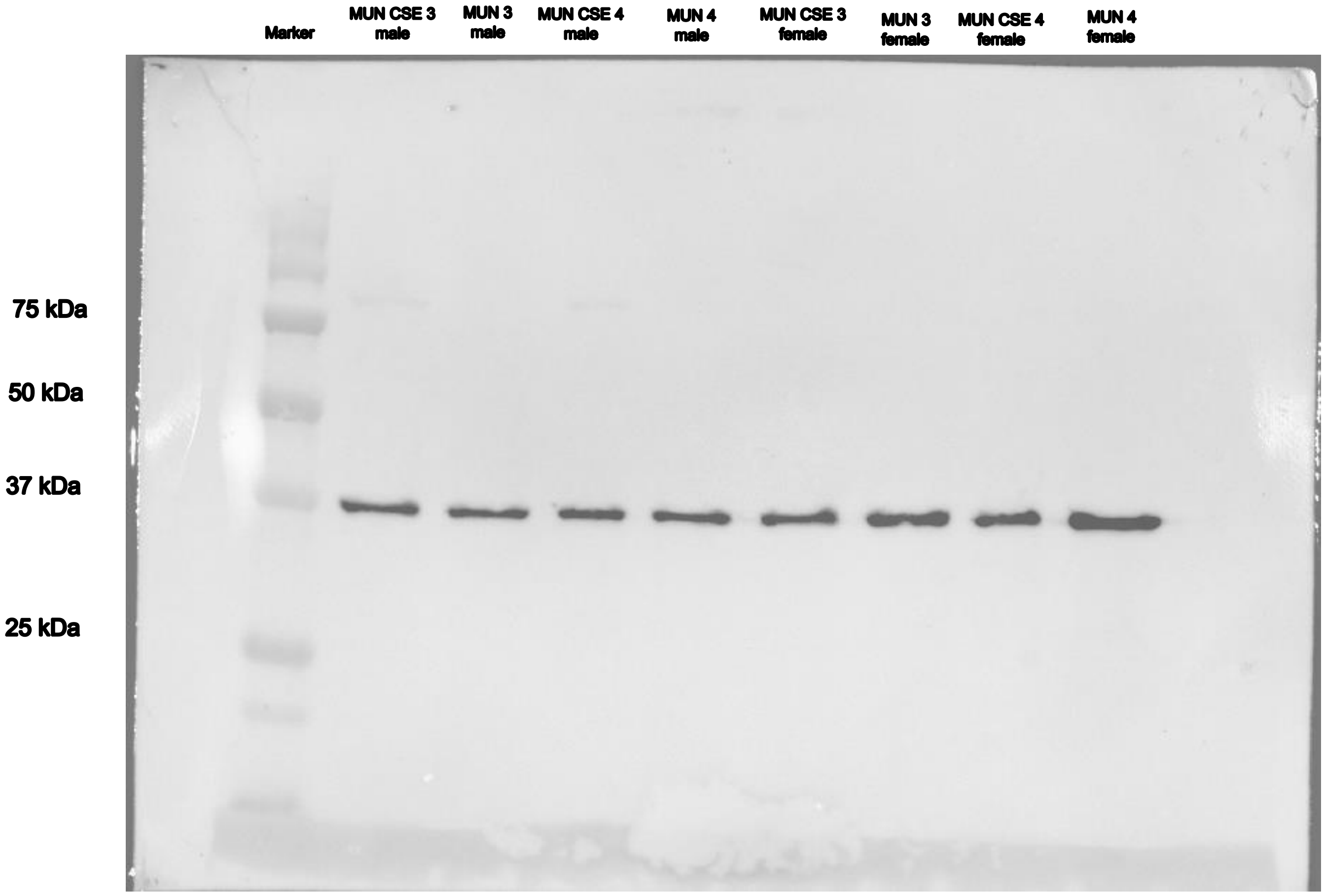

Marker
MUN CSE 3 male
MUN 3 male
MUN CSE 4 male
MUN 4 male
MUN CSE 3 female
MUN 3 female
MUN CSE 4 female
MUN 4 female
75 kDa
50 kDa
37 kDa
25 kDa

Heart. eNOS total

Gel 5

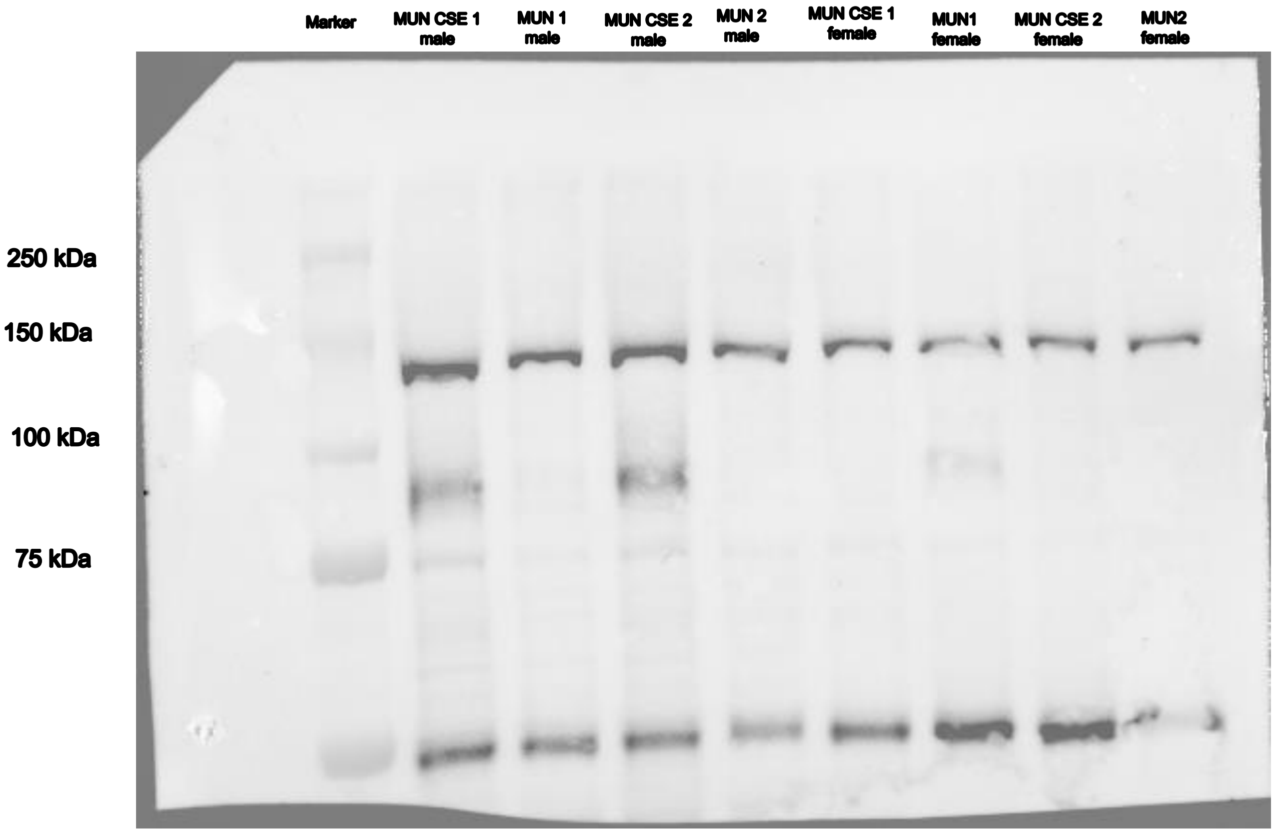

Gel 6

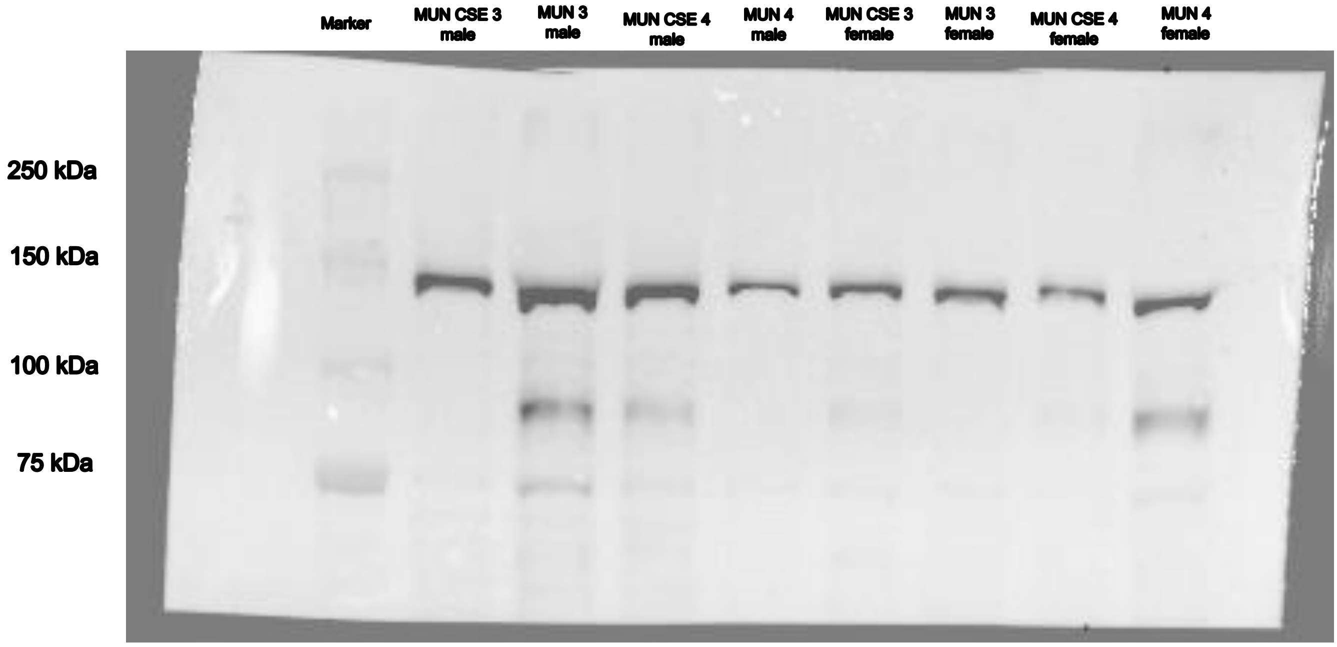
Marker
MUN CSE 3 male
MUN 3 male
MUN CSE 4 male
MUN 4 male
MUN CSE 3 female
MUN 3 female
MUN CSE 4 female
MUN 4 female
250 kDa
150 kDa
100 kDa
75 kDa

Heart. p-eNOS

Gel 5

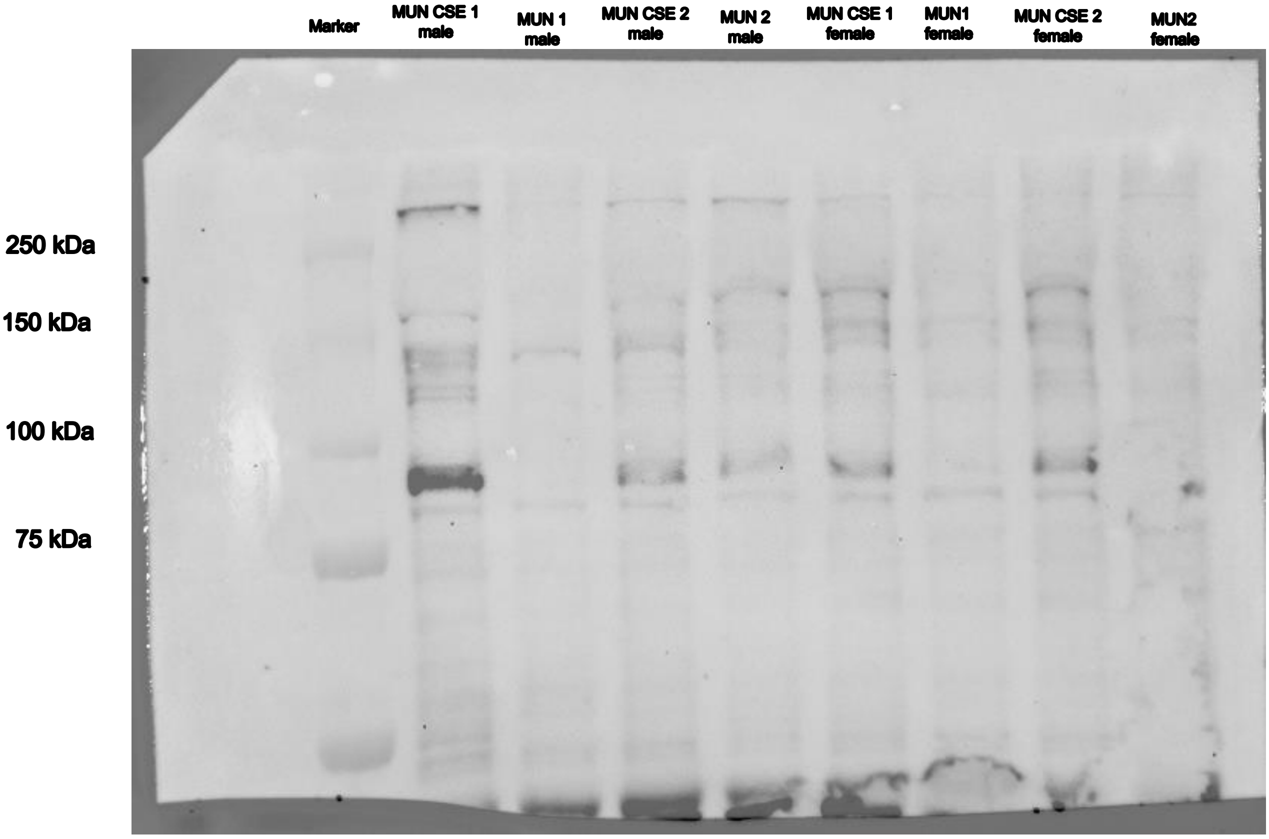

Gel 6

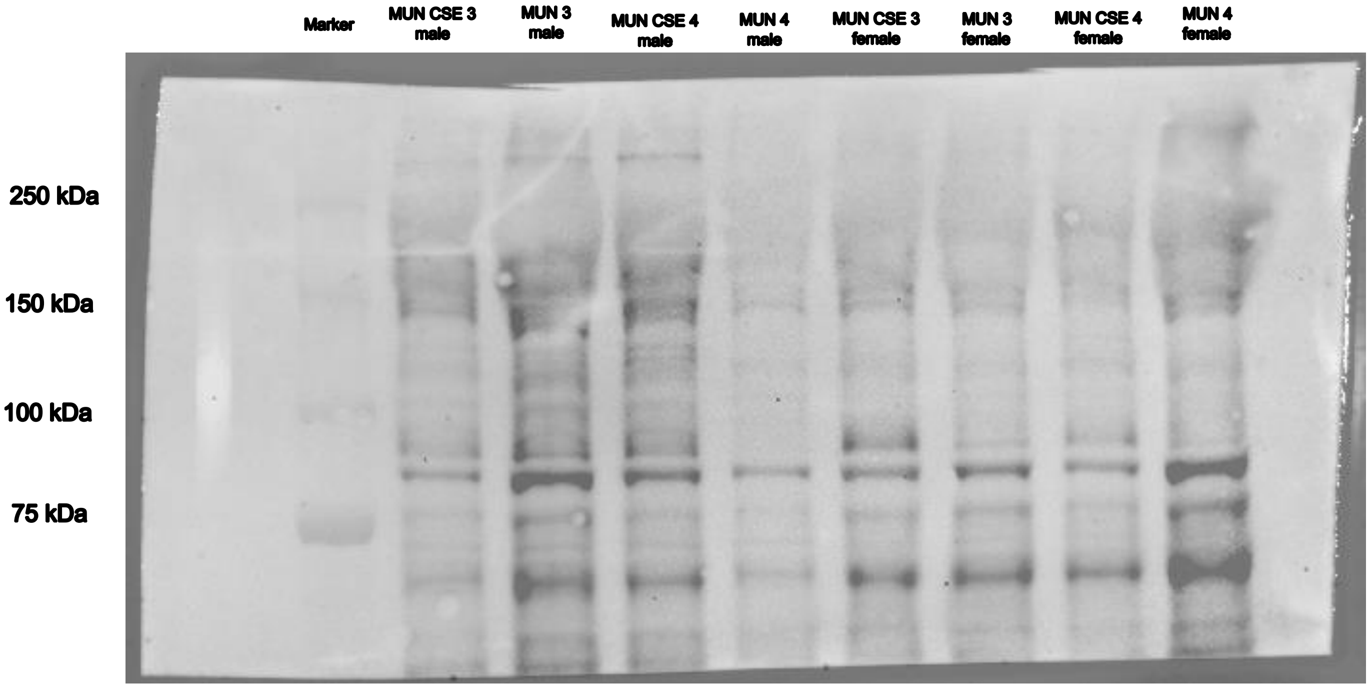

Marker
MUN CSE 3 male
MUN 3 male
MUN CSE 4 male
MUN 4 male
MUN CSE 3 female
MUN 3 female
MUN CSE 4 female
MUN 4 female
250 kDa
150 kDa
100 kDa
75 kDa

Heart. GADPH

Gel 5

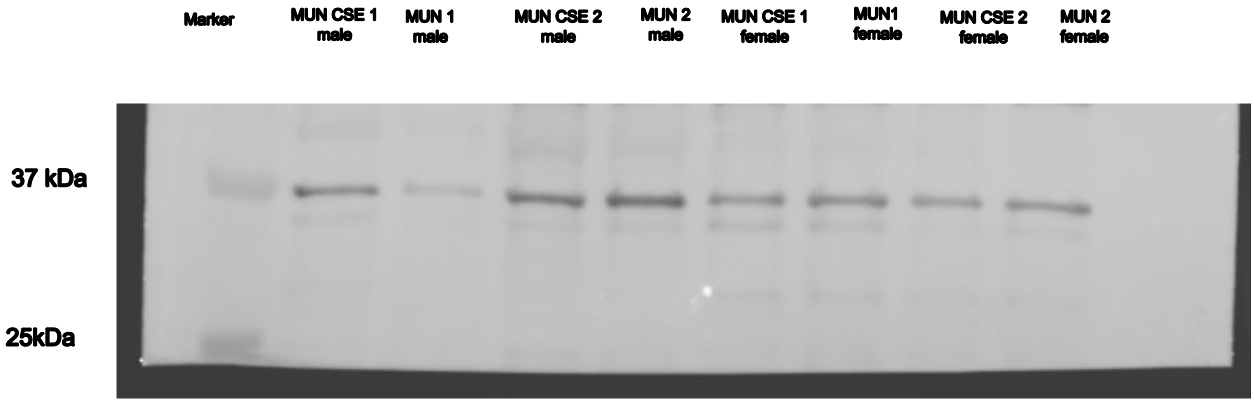

Gel 6

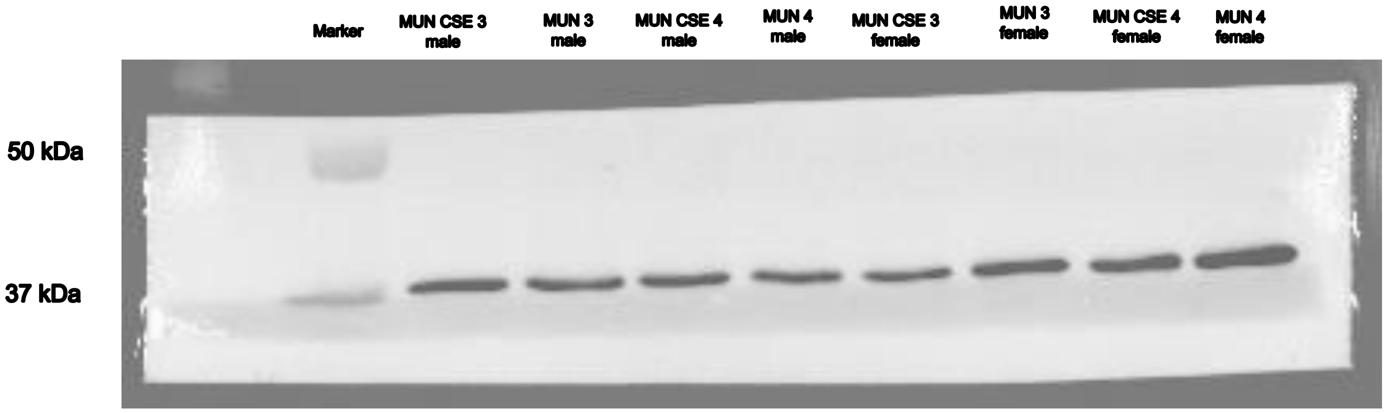

Aorta. eNOS total

Gel 7

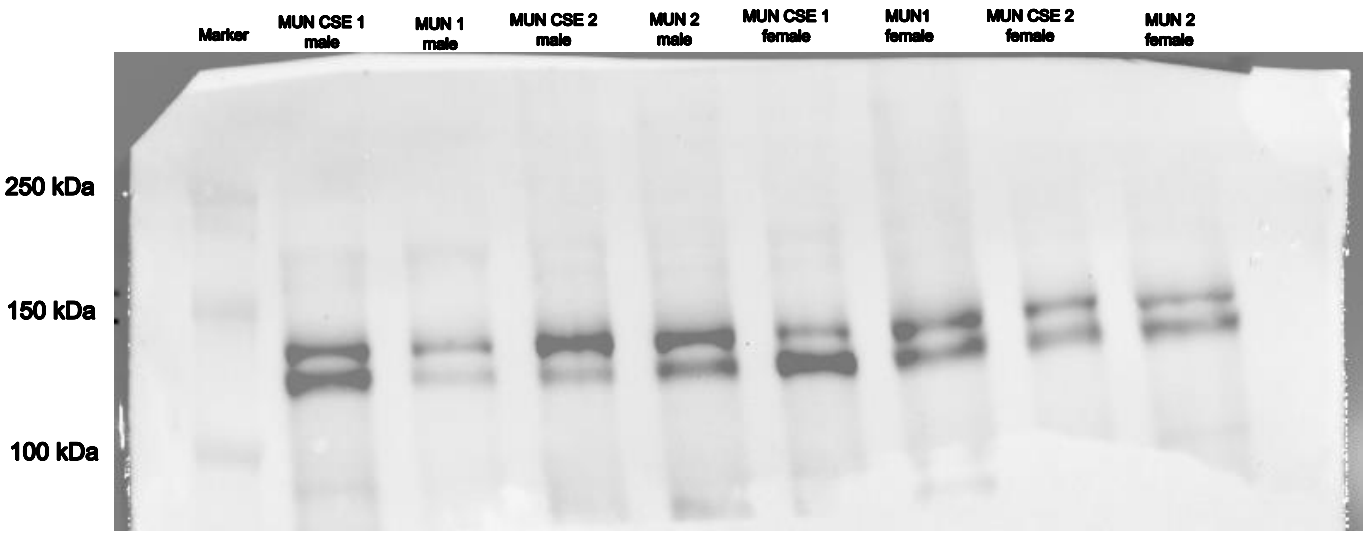

Gel 8

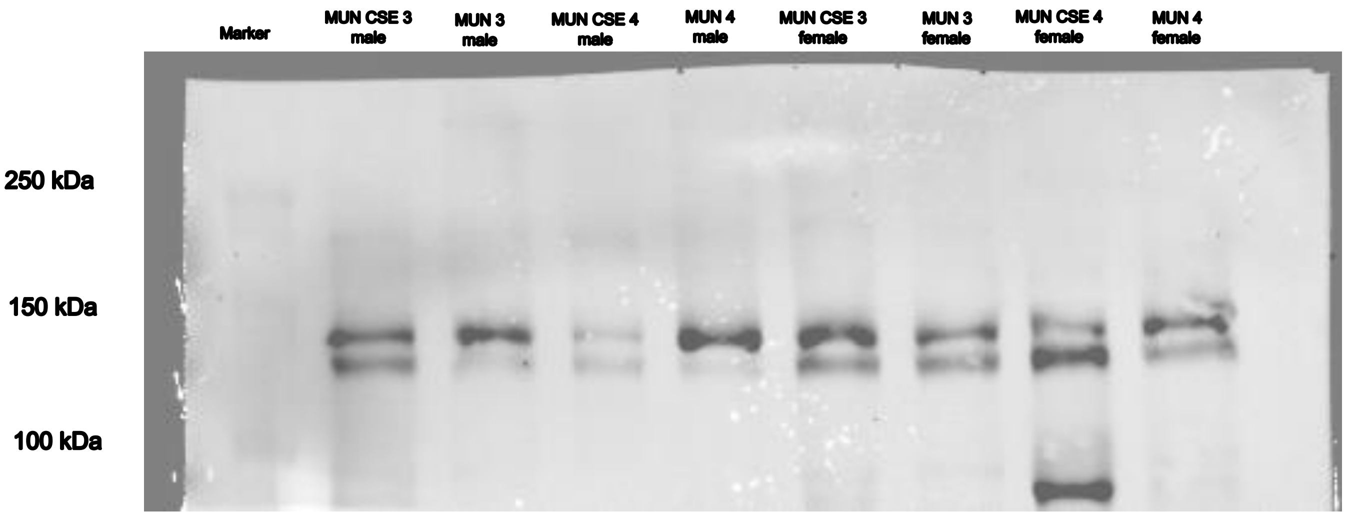

Aorta. GADPH

Gel 7

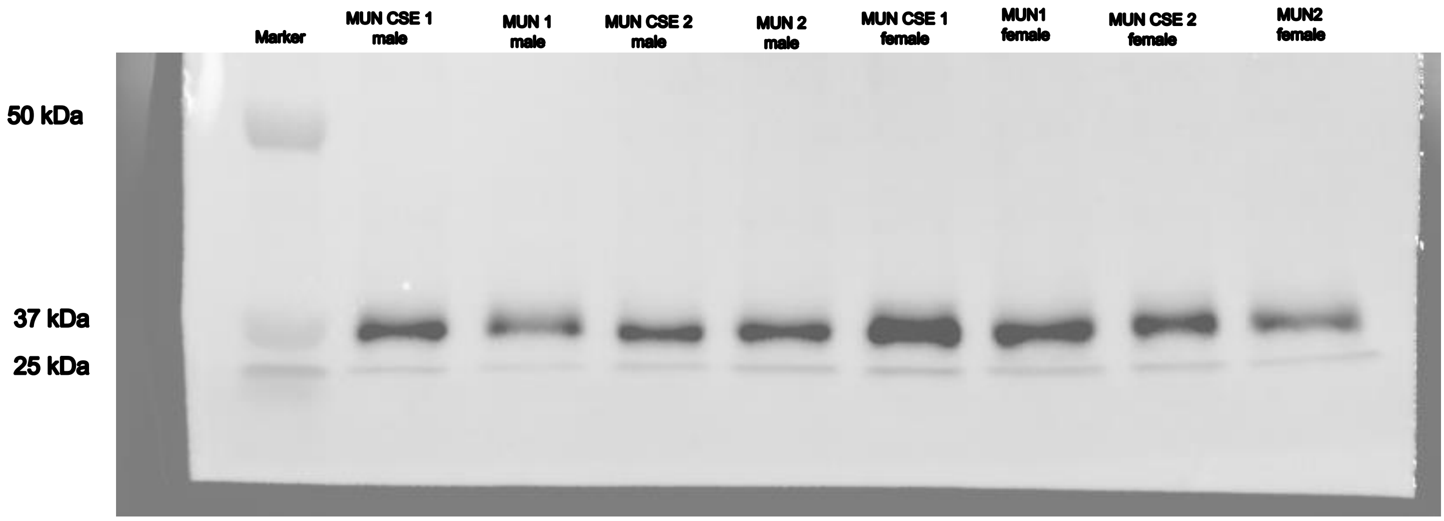

Gel 8

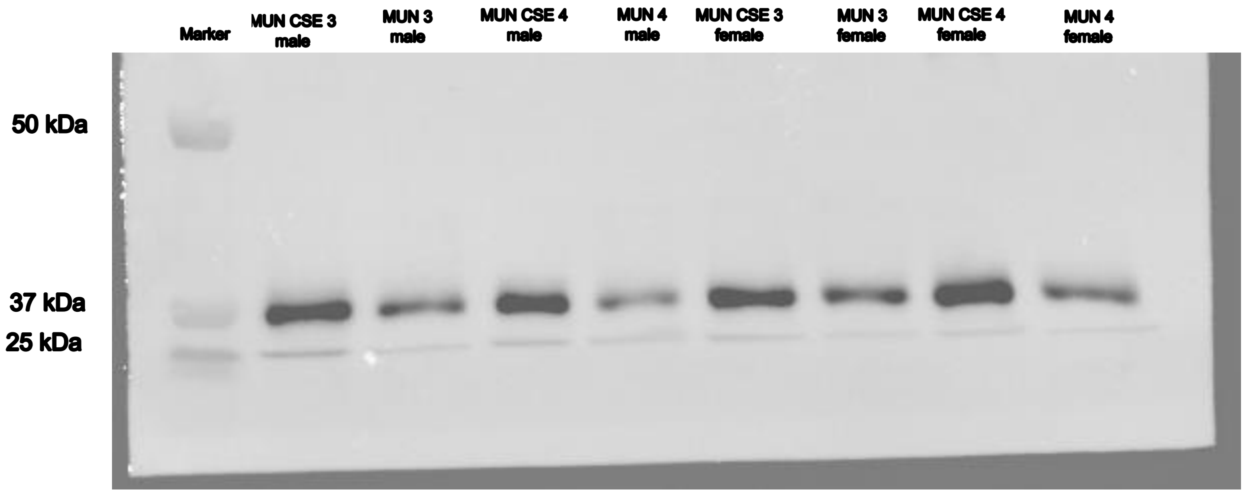

Heart. Catalase

Gel 9

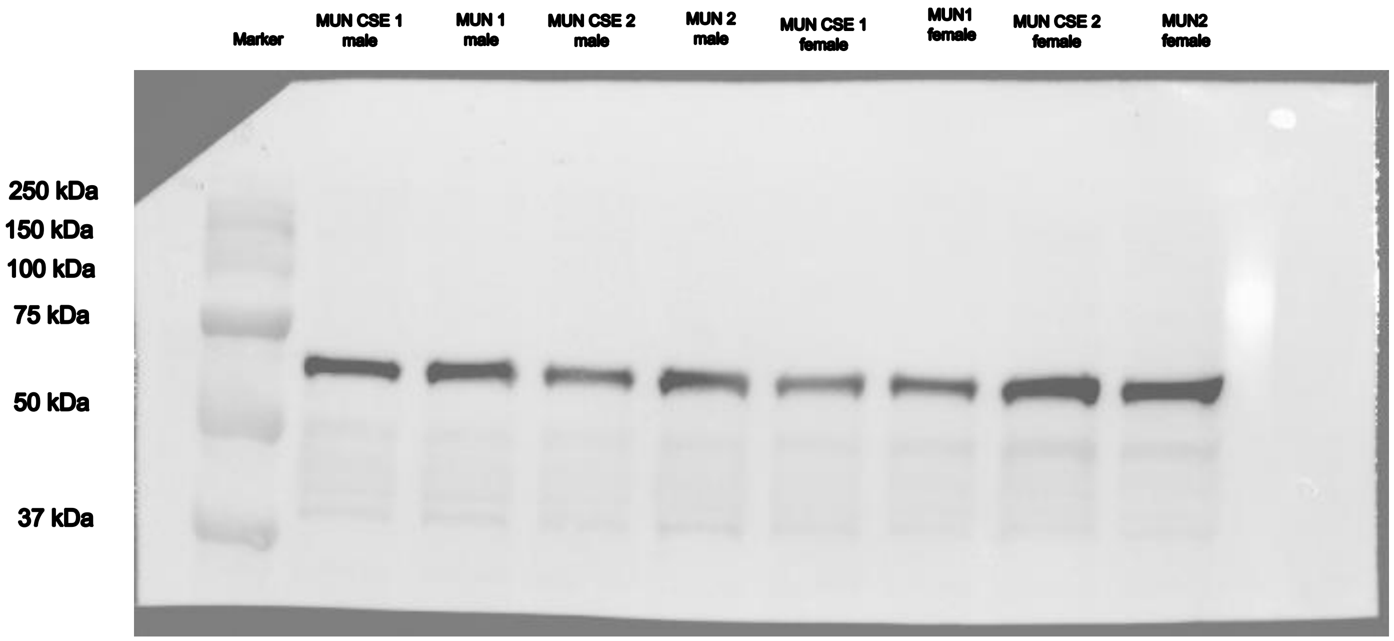

Gel 10

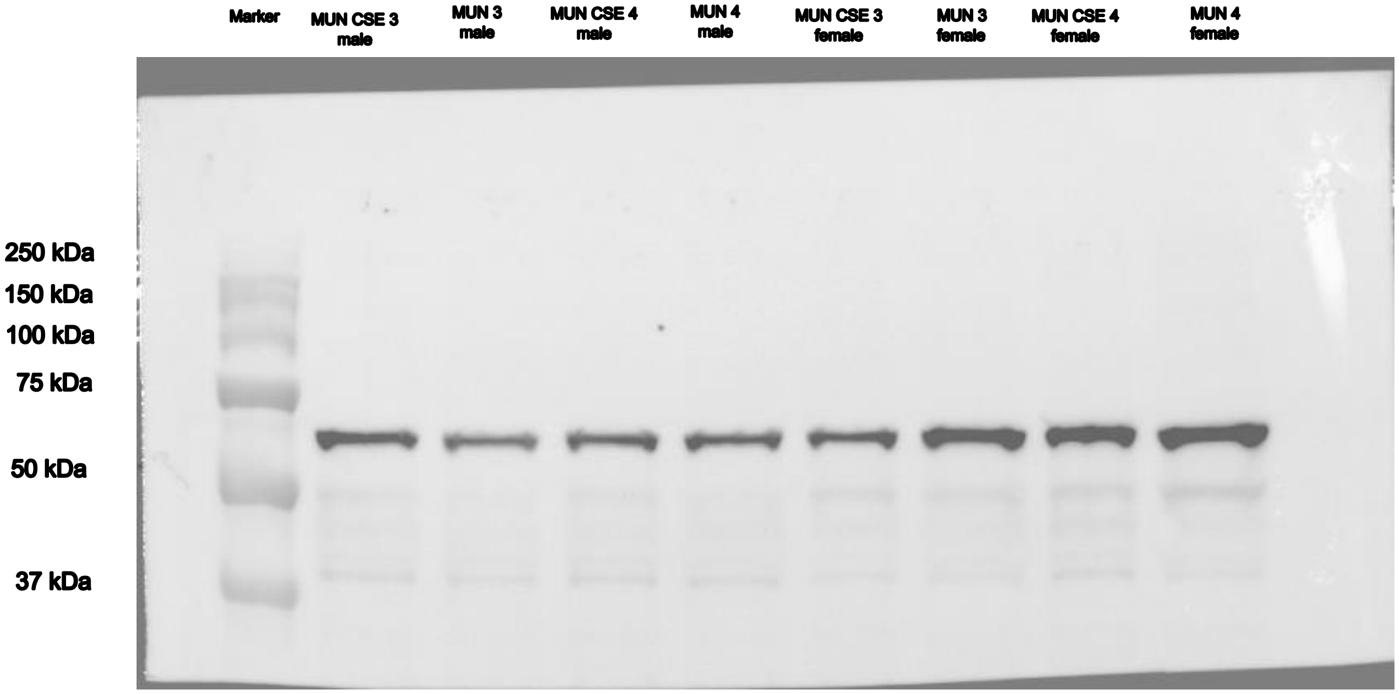

Heart. SOD 2

Gel 9

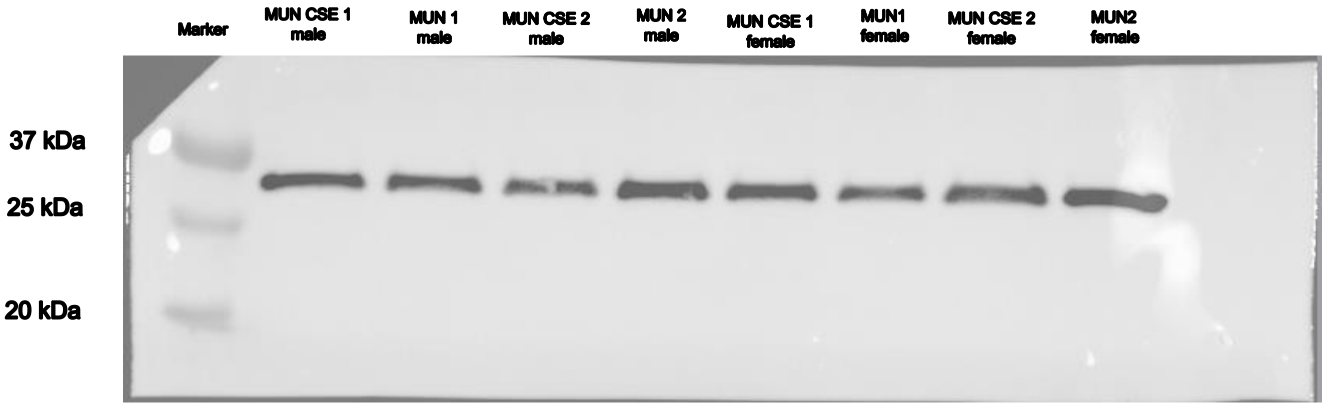

Gel 10

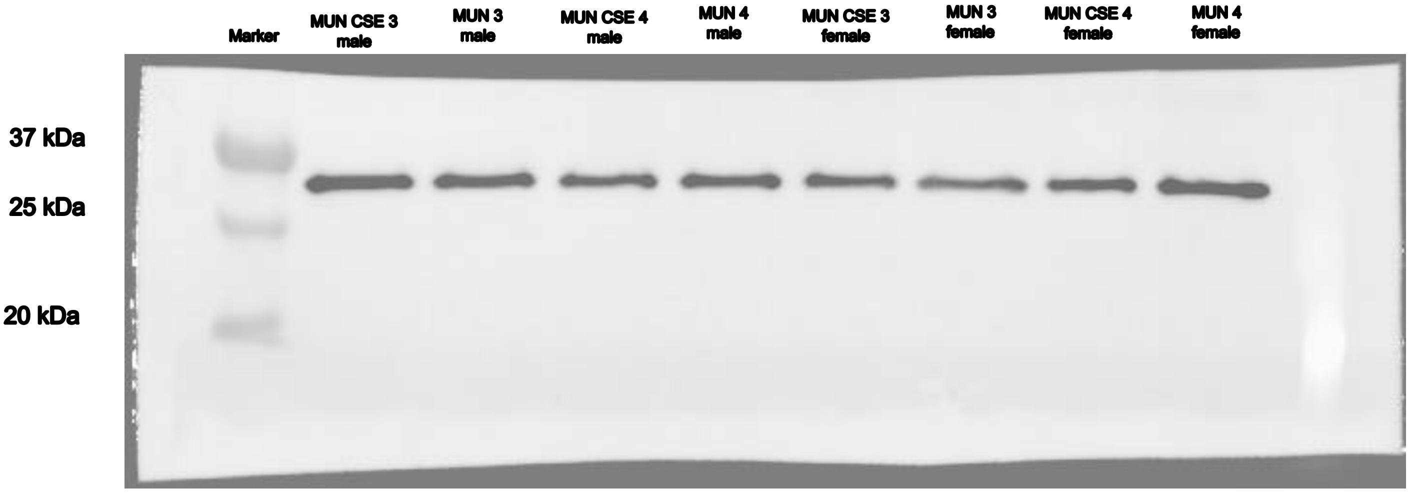

Heart. GAPDH

Gel 9

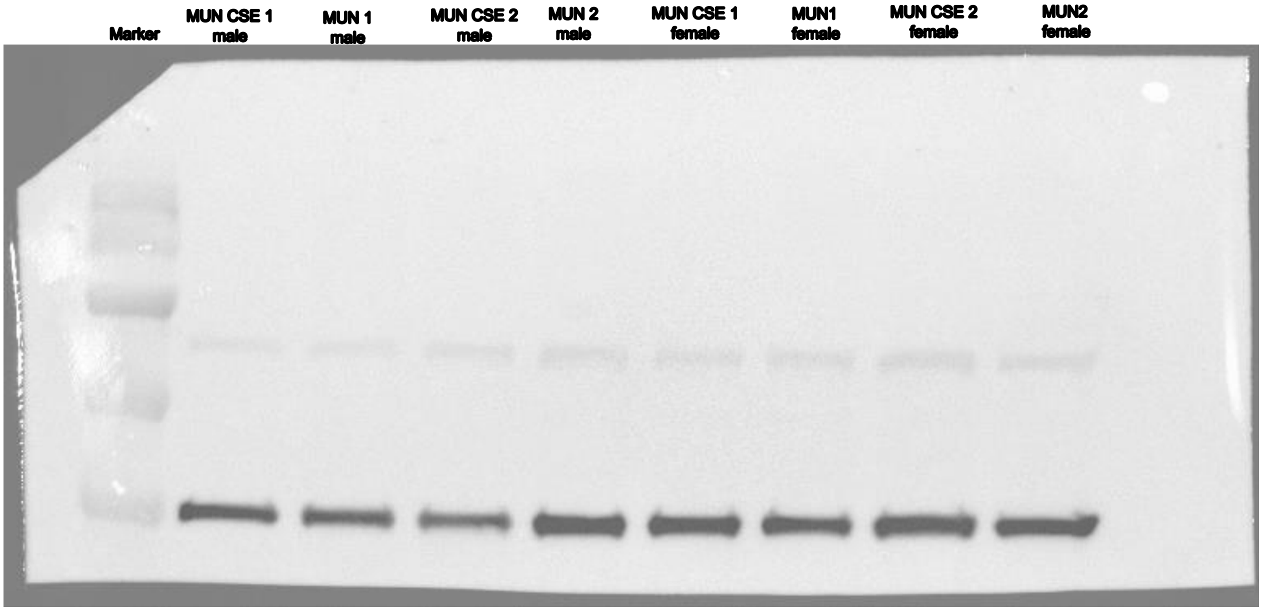

Gel 10

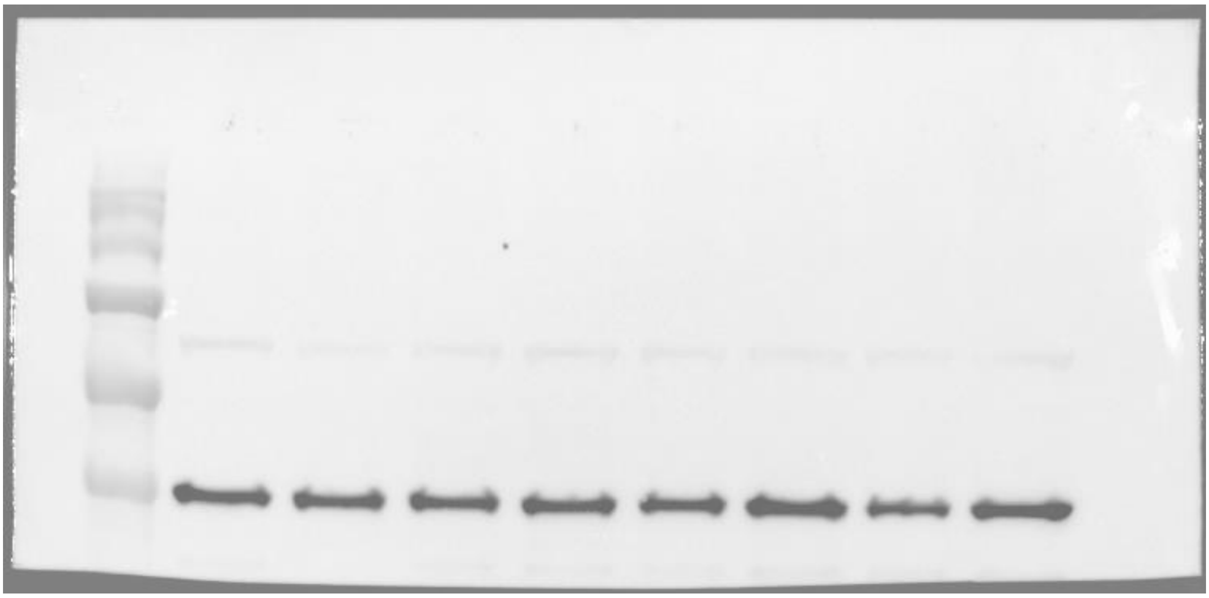

Heart. HO-1

Gel 11

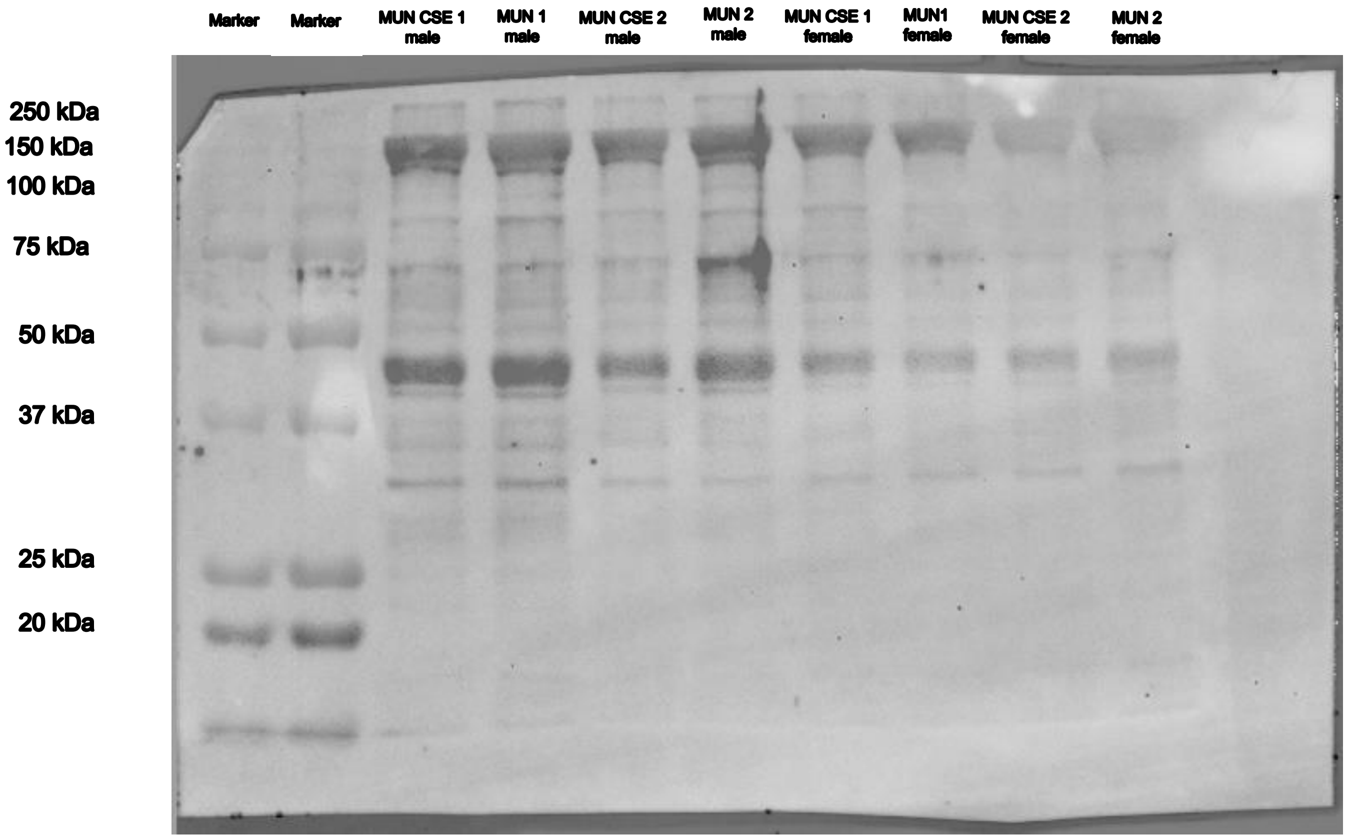

Gel 12

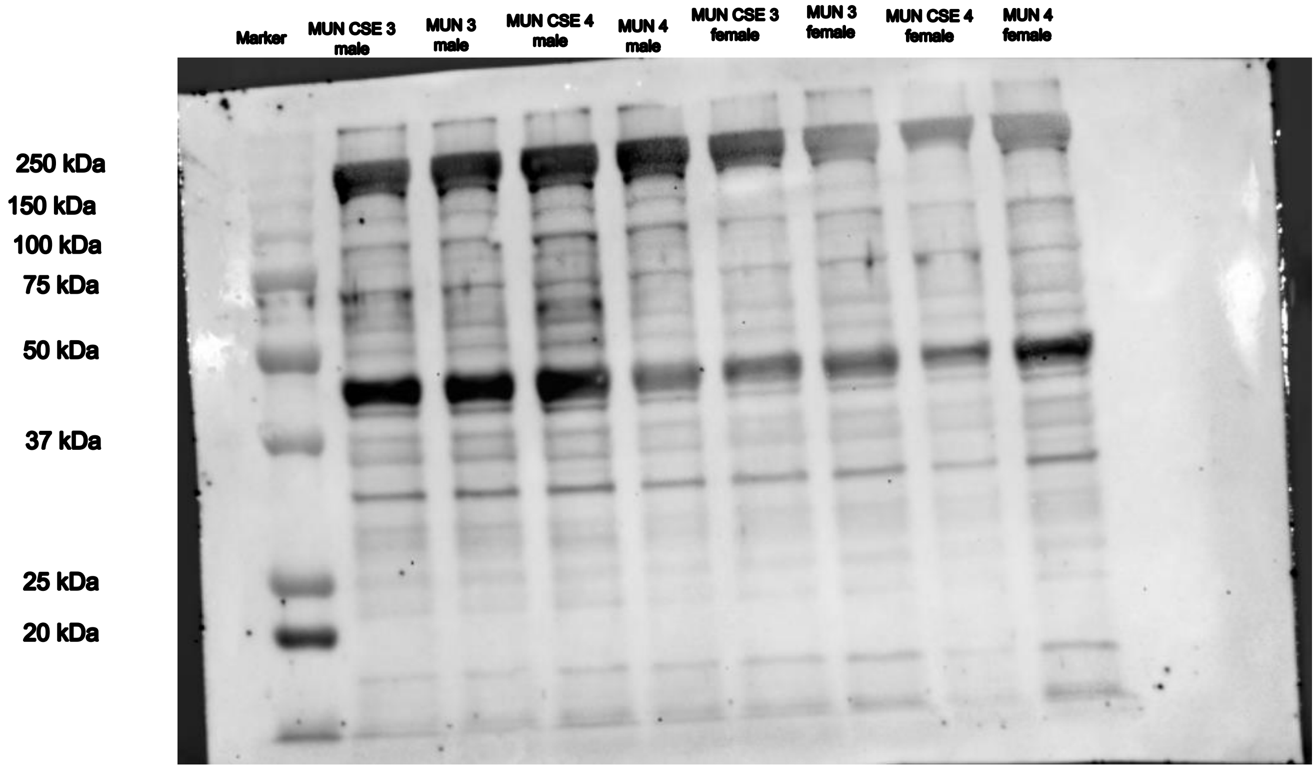

Heart. UCP-2

Gel 11

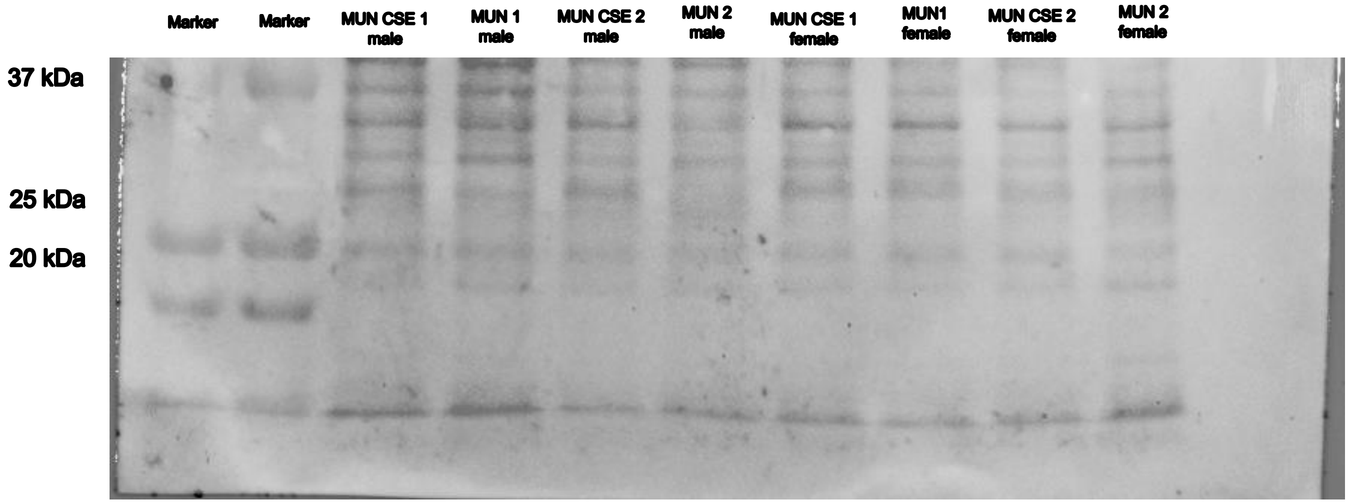

Gel 12

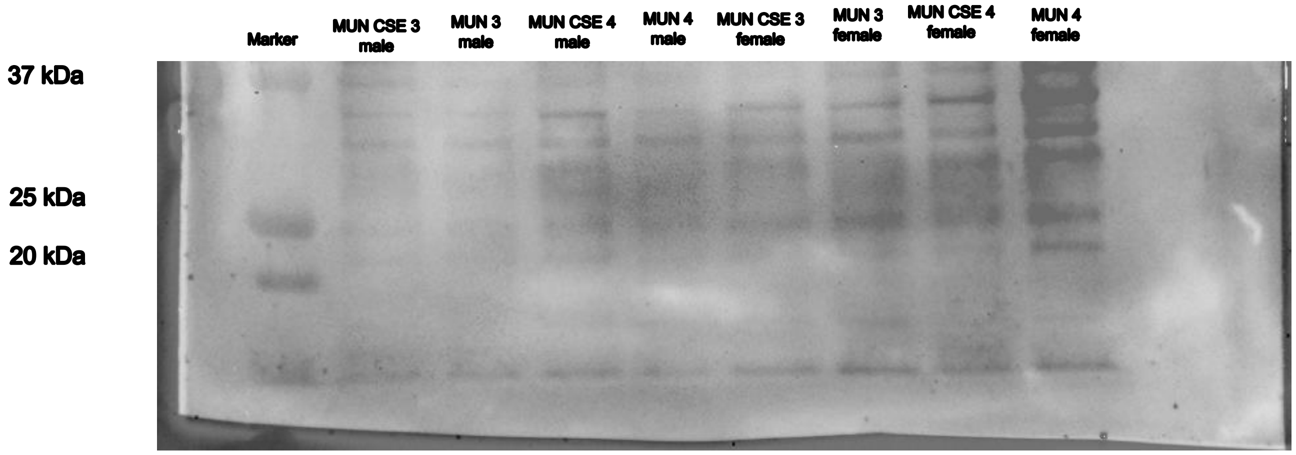

Heart. GAPDH

Gel 11

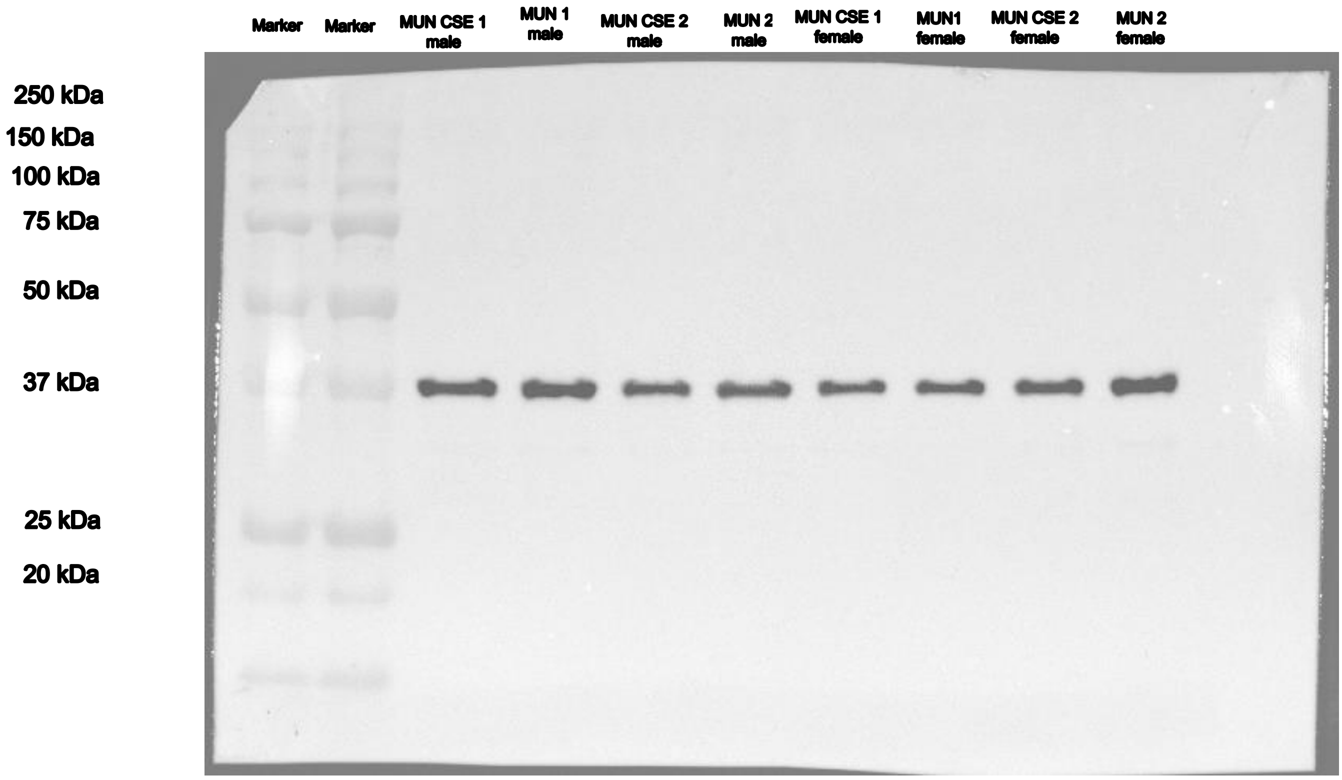

Gel 12

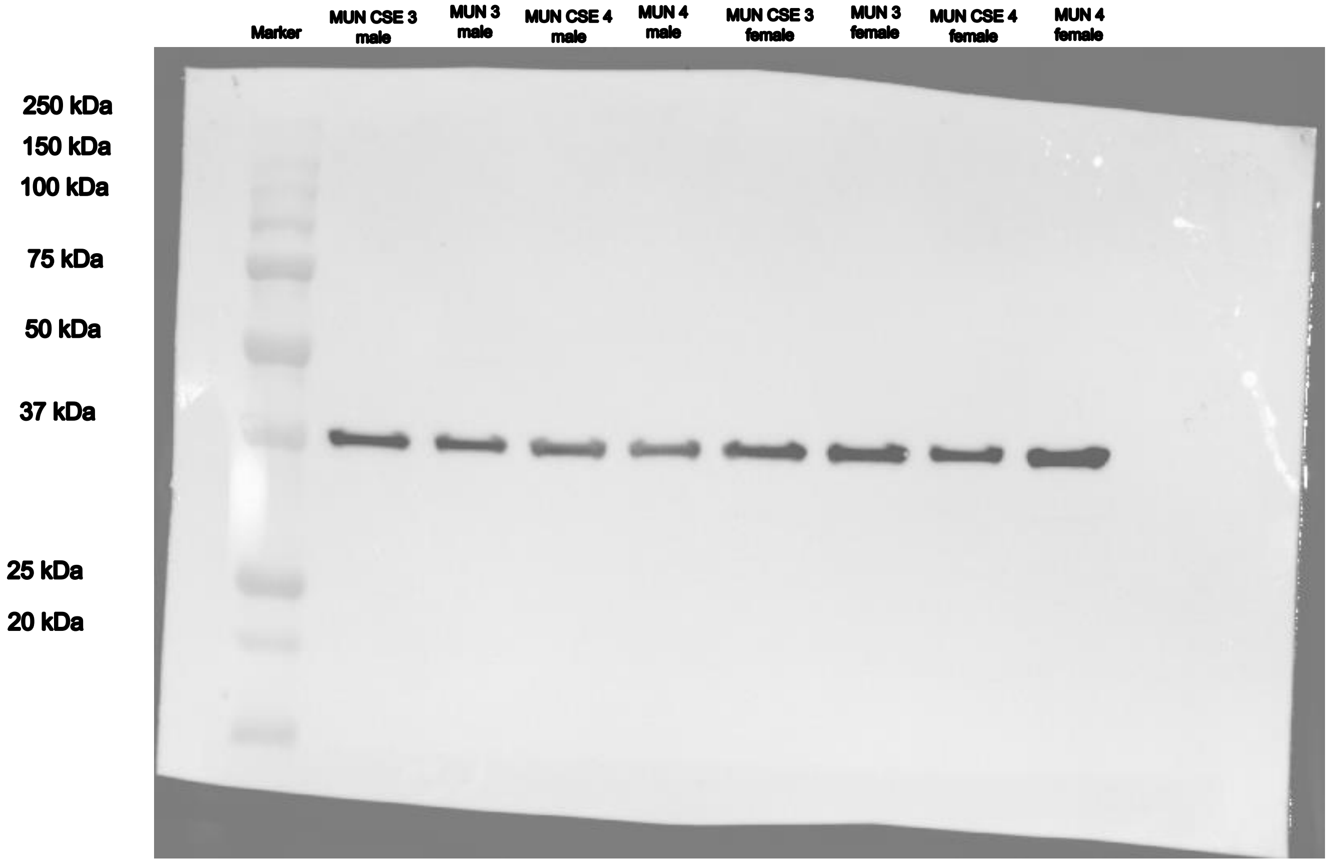
Marker
MUN CSE 3 male
MUN 3 male
MUN CSE 4 male
MUN 4 male
MUN CSE 3 female
MUN 3 female
MUN CSE 4 female
MUN 4 female
250 kDa
150 kDa
100 kDa
75 kDa
50 kDa
37 kDa
25 kDa
20 kDa

Aorta. Catalase

Gel 13

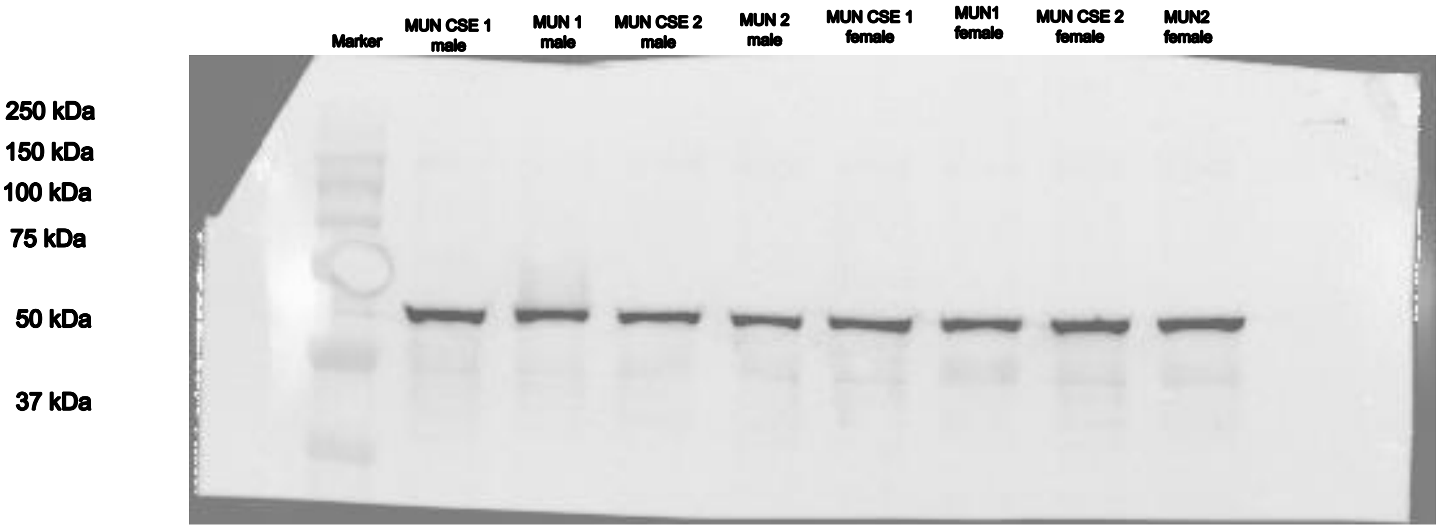

Gel 14

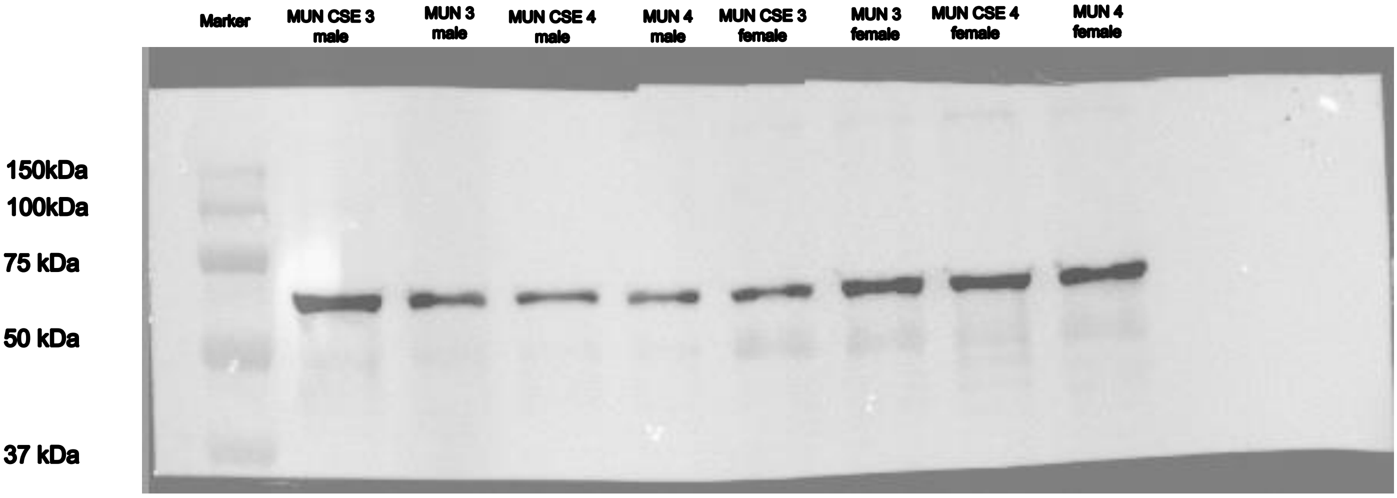

Aorta. SOD-2

Gel 13

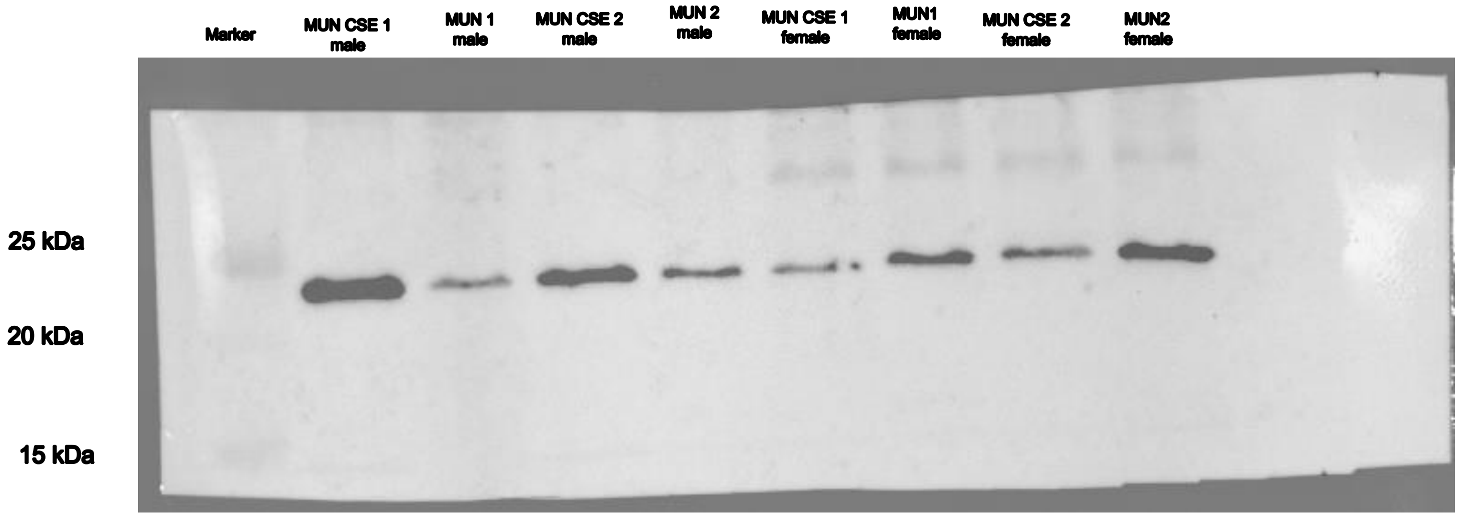

Gel 14

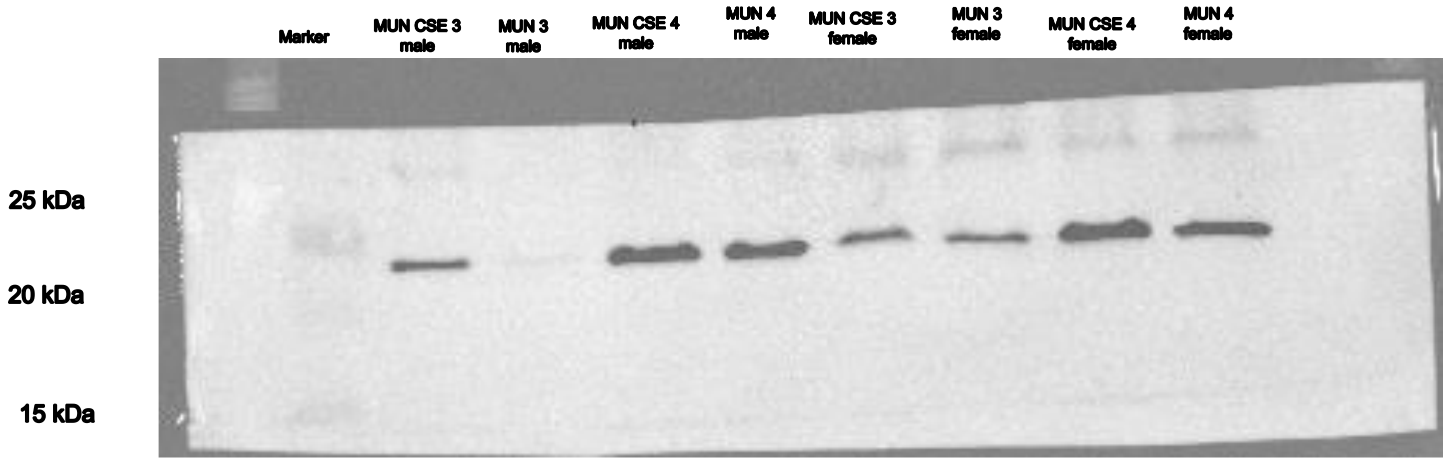

Aorta. GAPDH

Gel 13

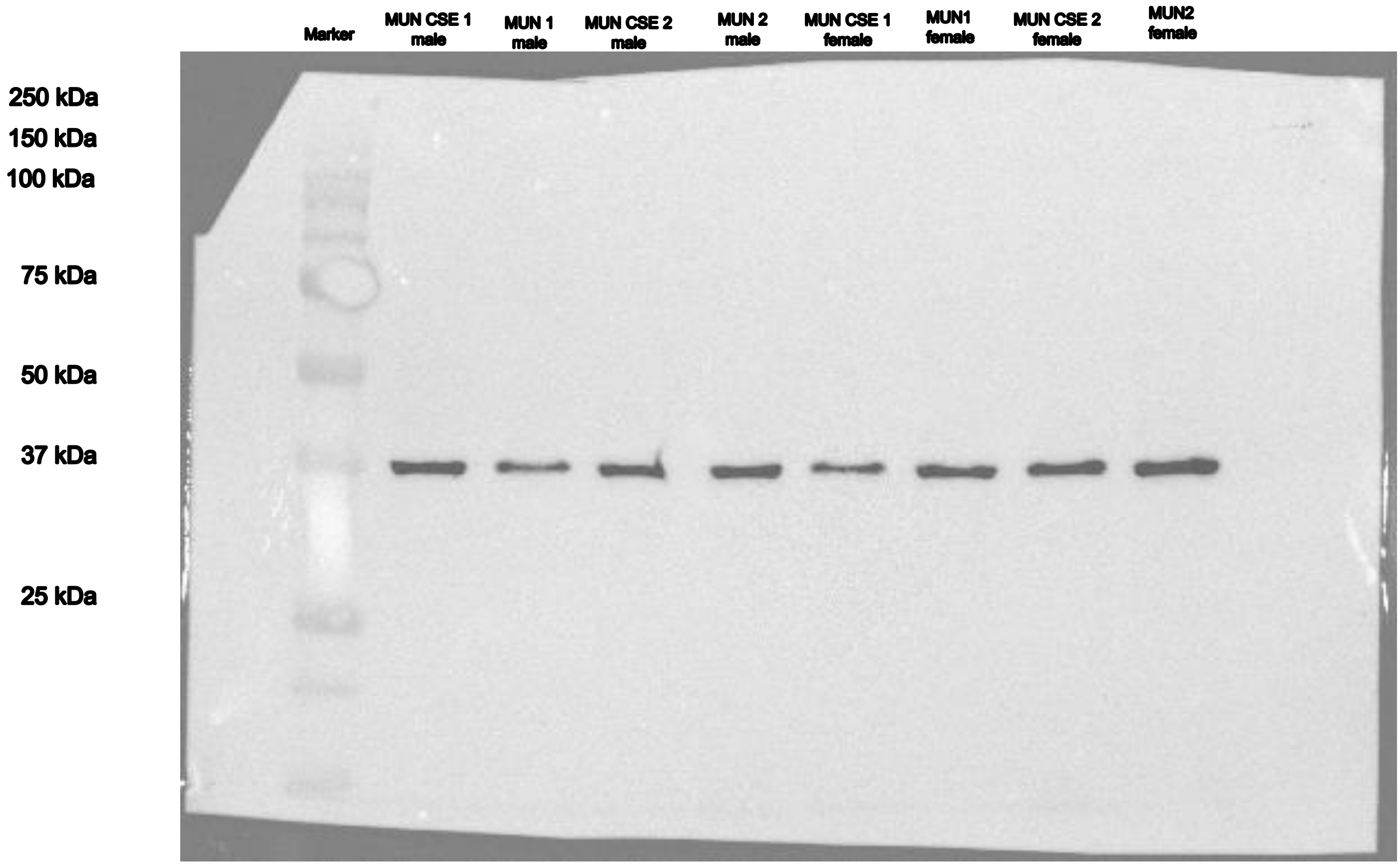

Gel 14

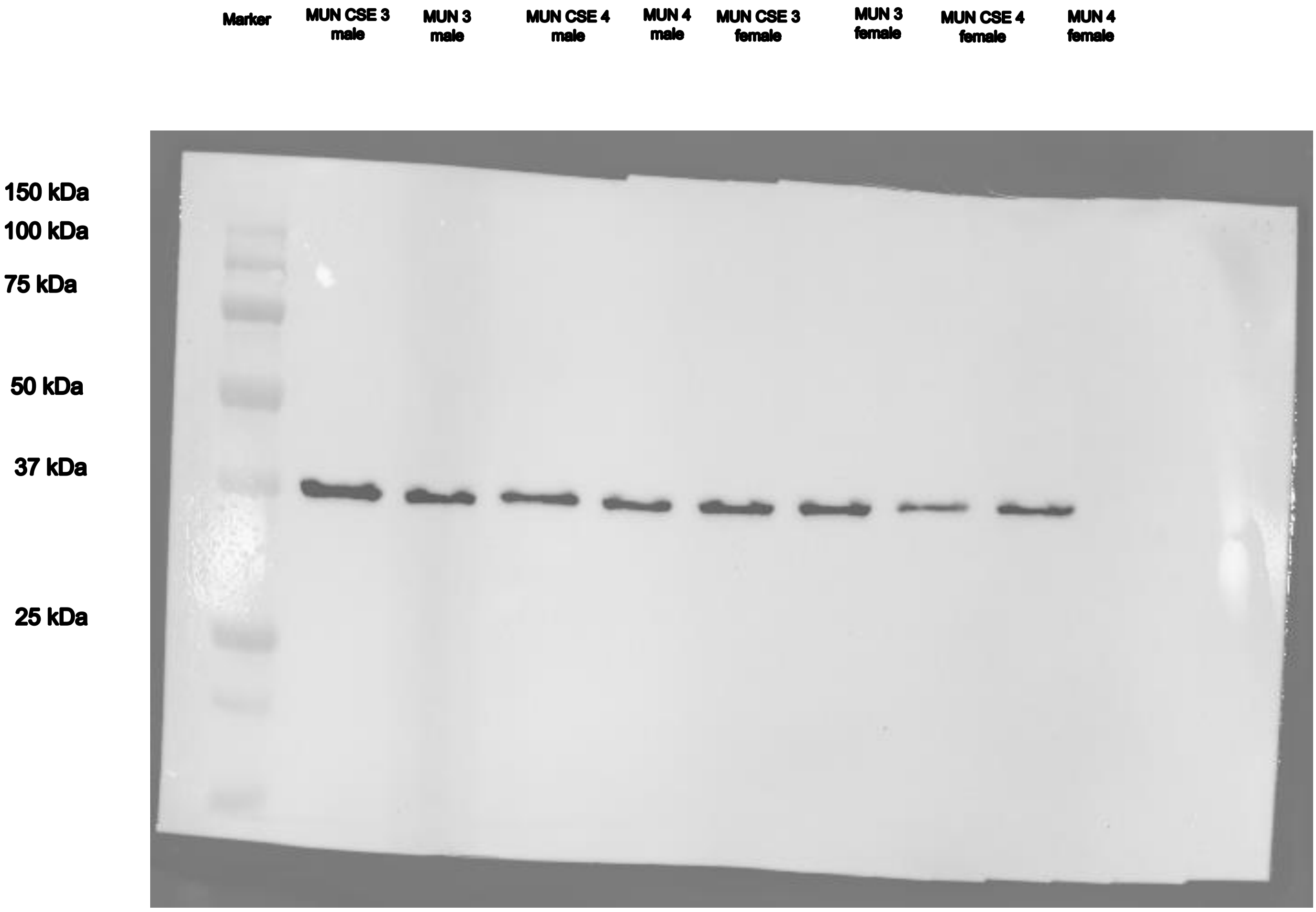

Marker
MUN CSE 3 male
MUN 3 male
MUN CSE 4 male
MUN 4 male
MUN CSE 3 female
MUN 3 female
MUN CSE 4 female
MUN 4 female
150 kDa
100 kDa
75 kDa
50 kDa
37 kDa
25 kDa

Aorta. HO-1

Gel 15

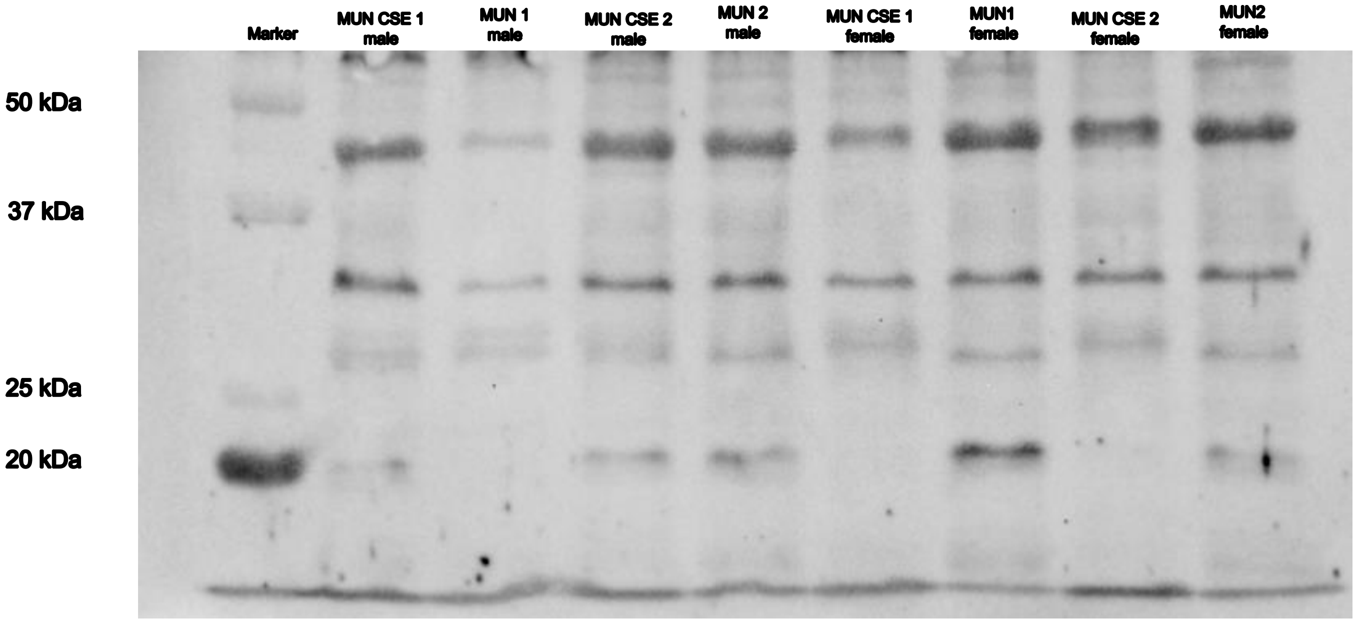

Gel 16

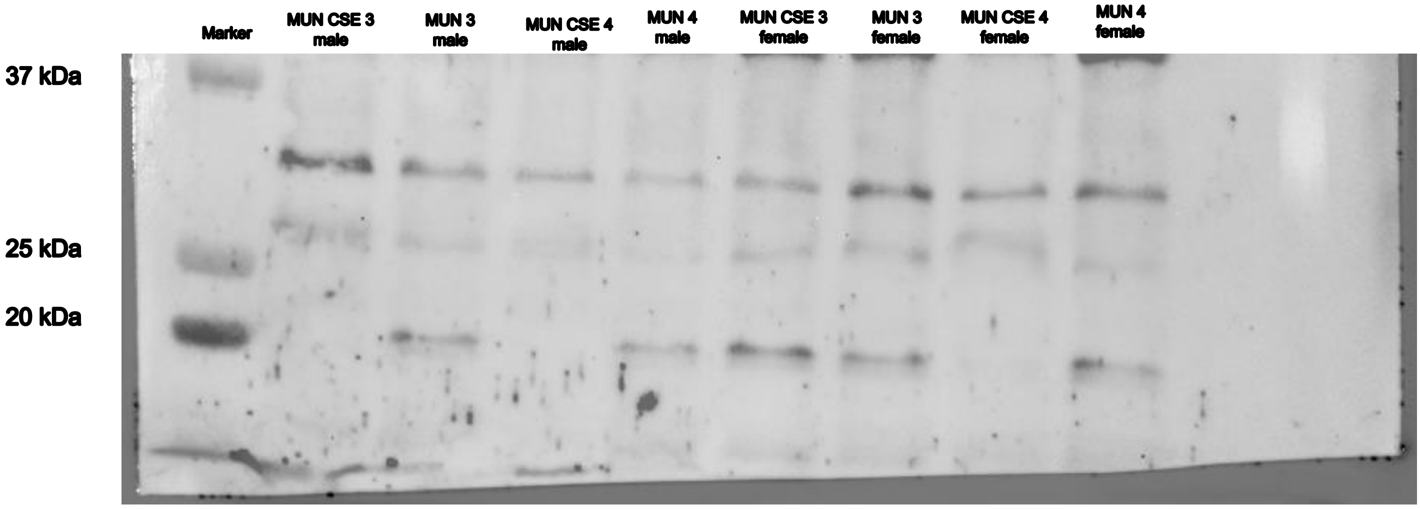

Aorta. GAPDH

Gel 15

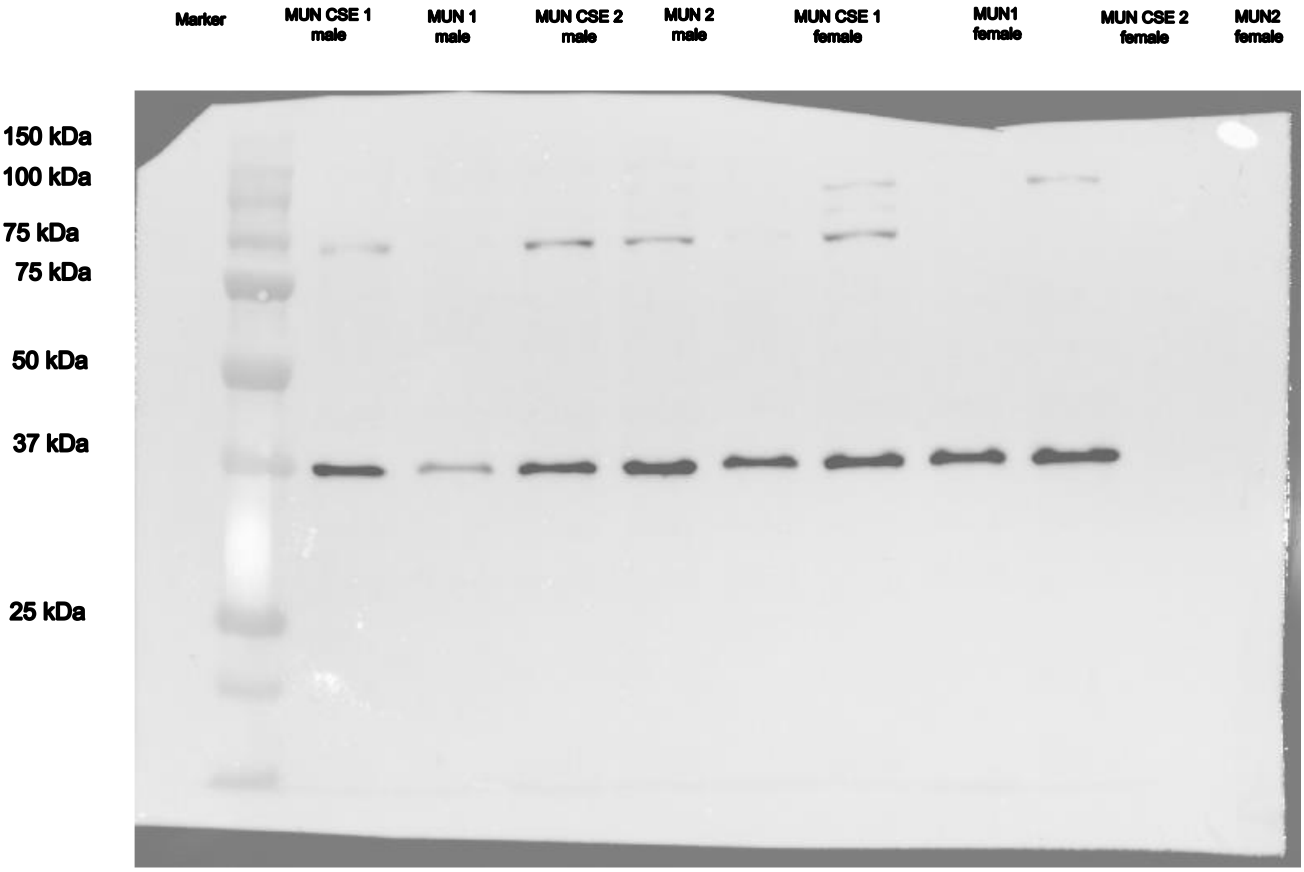

Gel 16

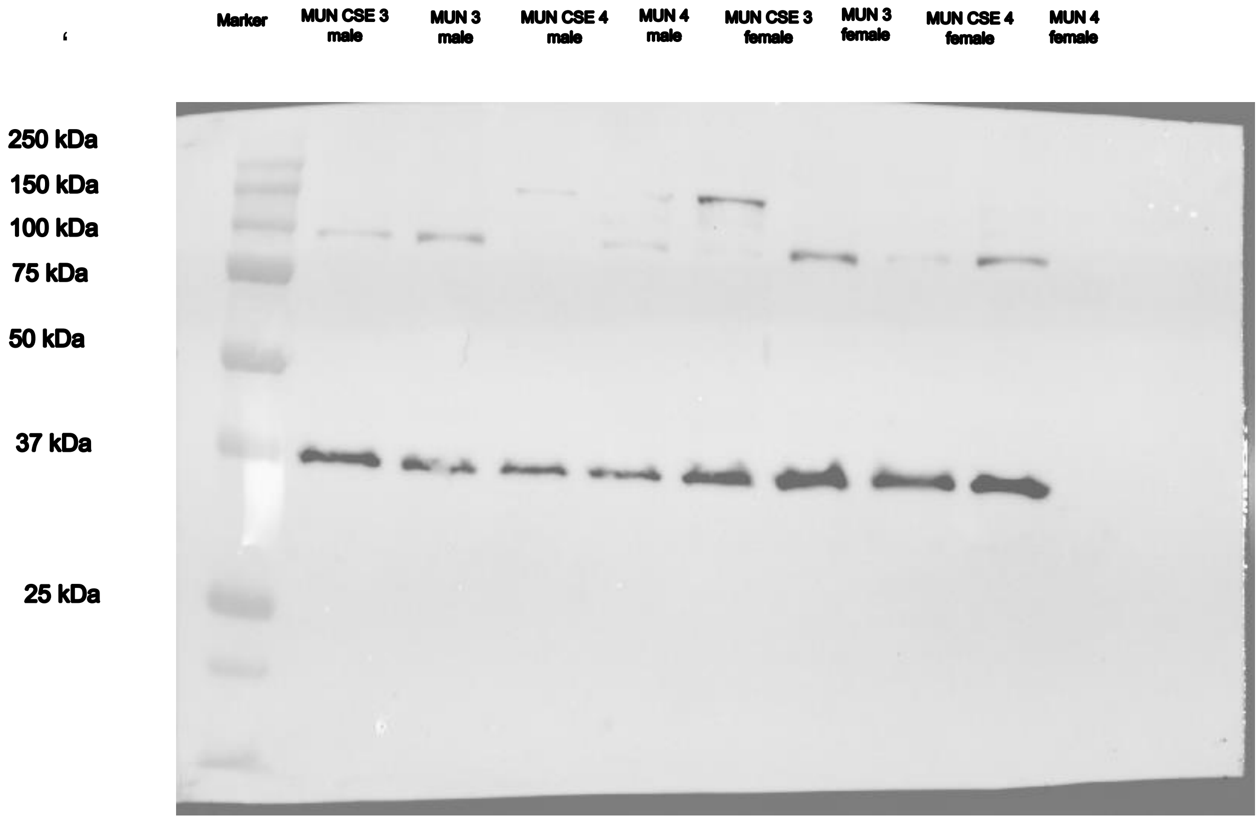

Aorta. UCP-2

Gel 17

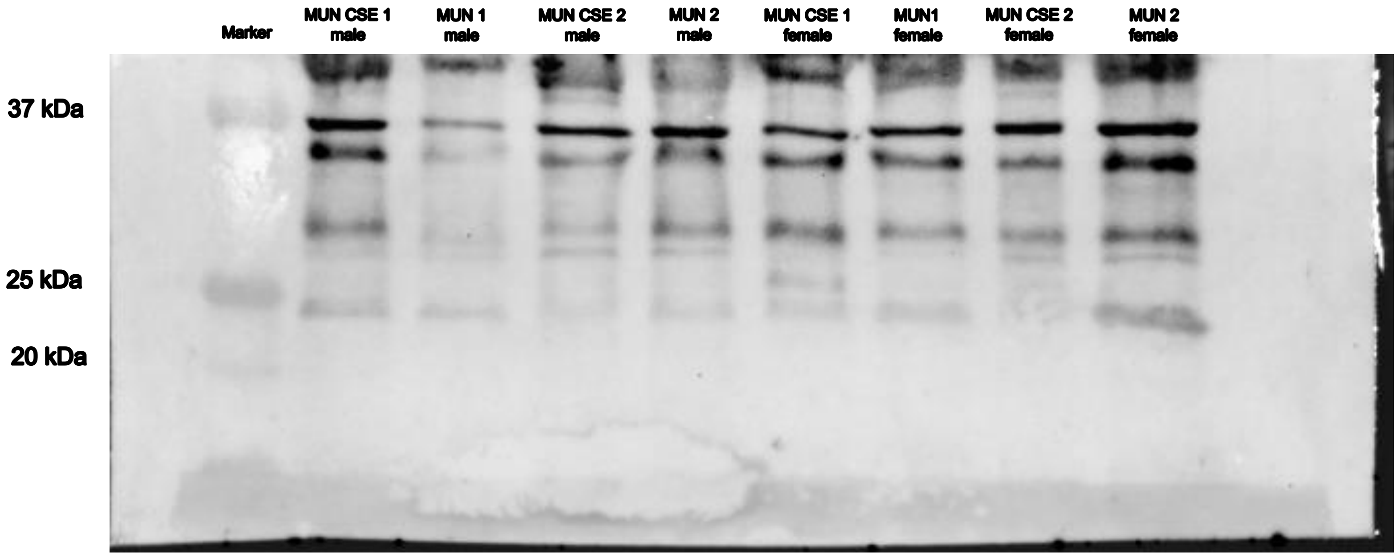

Gel 18

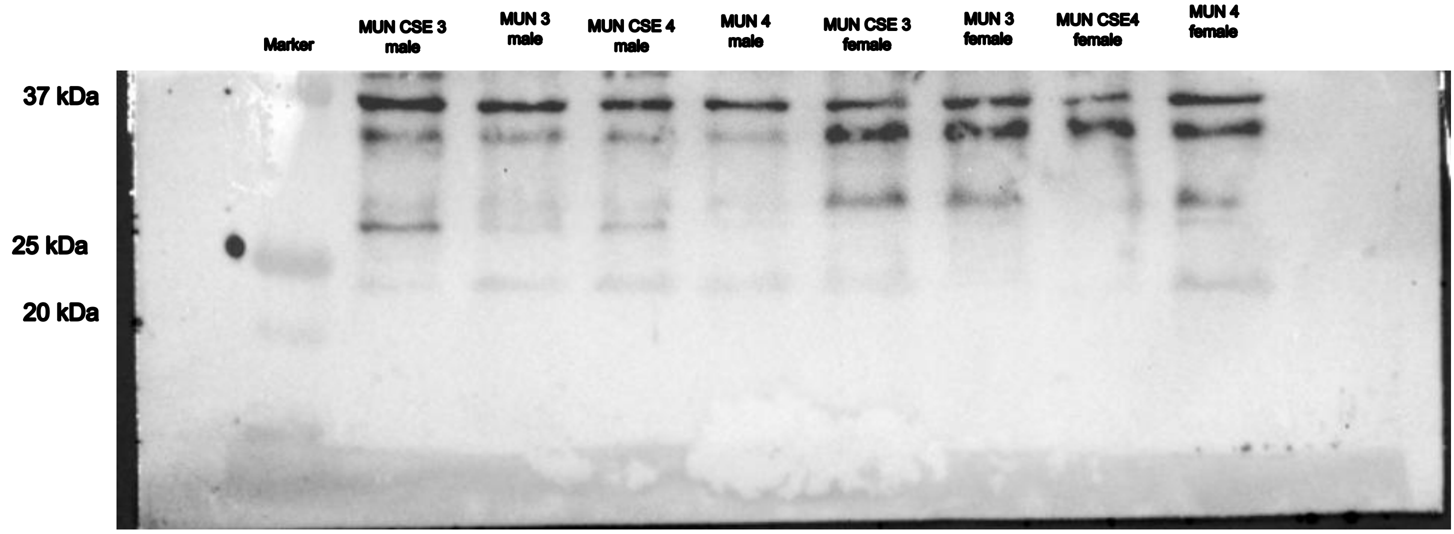

GADPH

Gel 17

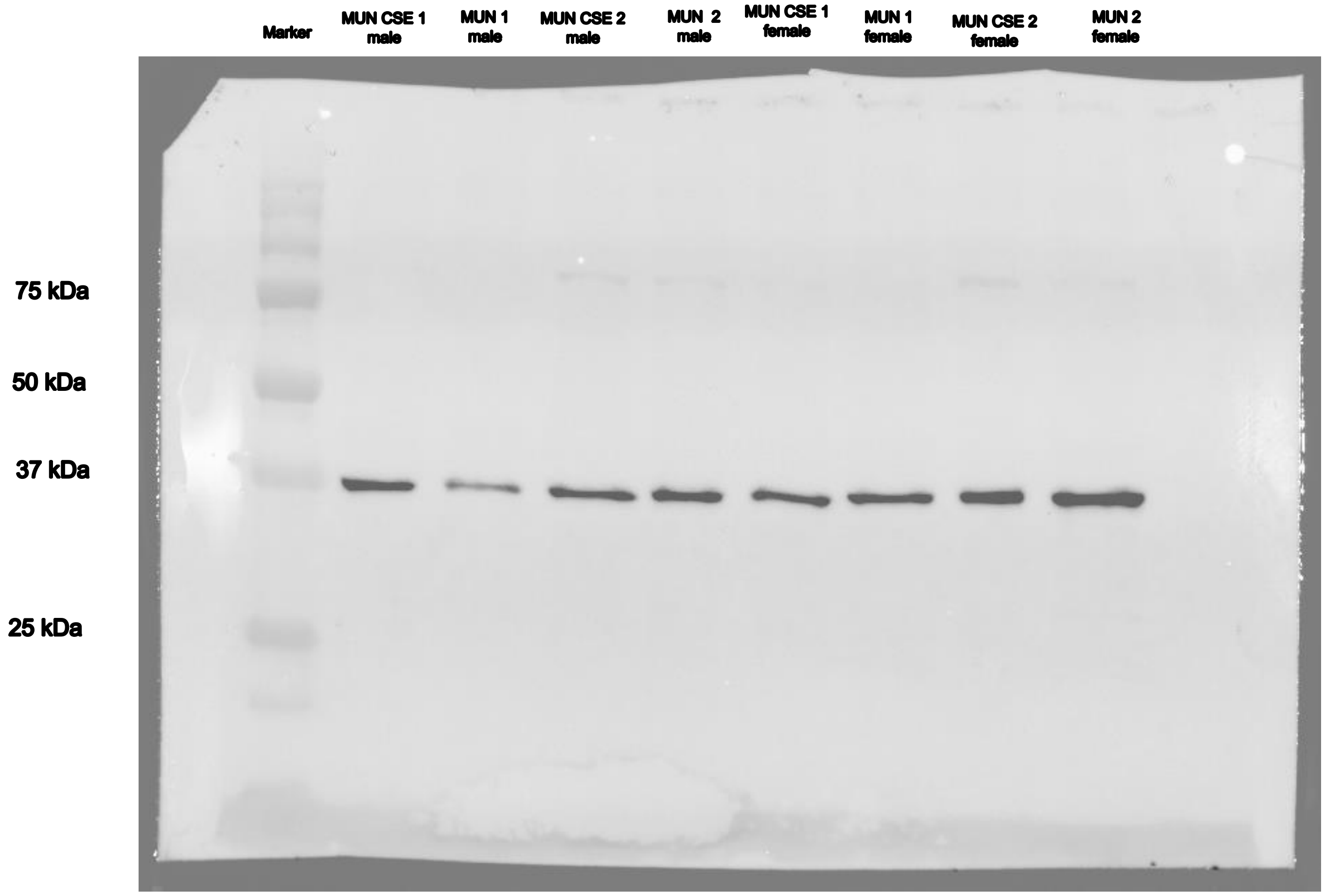

Gel 18

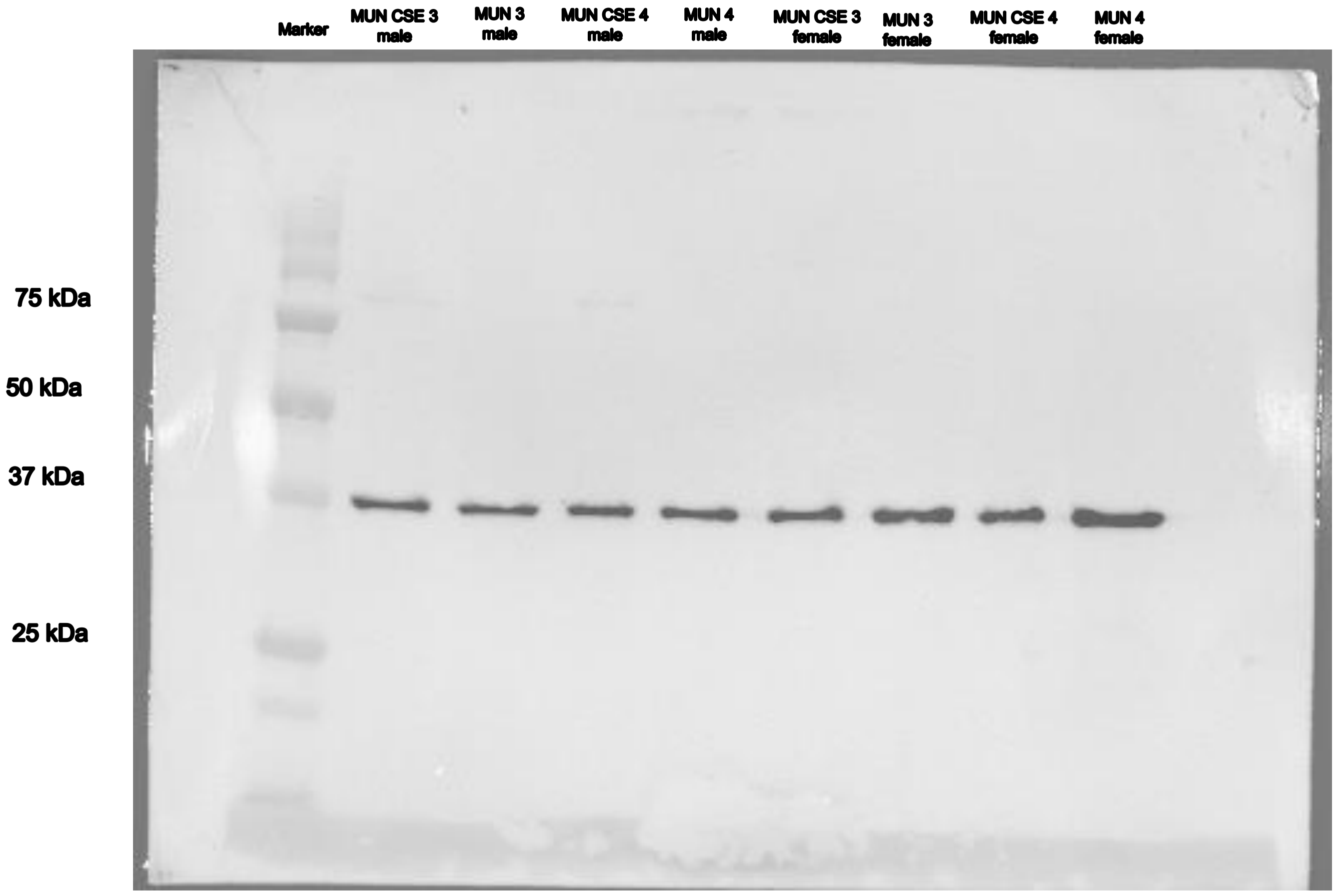
Marker
MUN CSE 3 male
MUN 3 male
MUN CSE 4 male
MUN 4 male
MUN CSE 3 female
MUN 3 female
MUN CSE 4 female
MUN 4 female
75 kDa
50 kDa
37 kDa
25 kDa
